# Homochiral Mn^3+^ Spin-Crossover Complexes: A Structural and Spectroscopic Study

**DOI:** 10.1021/acs.inorgchem.1c03379

**Published:** 2022-02-17

**Authors:** Irina A. Kühne, Andrew Ozarowski, Aizuddin Sultan, Kane Esien, Anthony B. Carter, Paul Wix, Aoife Casey, Mooneerah Heerah-Booluck, Tony D. Keene, Helge Müller-Bunz, Solveig Felton, Stephen Hill, Grace G. Morgan

**Affiliations:** † School of Chemistry, University College Dublin (UCD), Belfield, Dublin 4, Ireland; ‡ FZU - Institute of Physics - Czech Academy of Sciences, Na Slovance 1999/2, Prague 8 182 21, Czech Republic; § National High Magnetic Field Laboratory, Florida State University, Tallahassee, Florida 32310, United States; ∥ School of Mathematics and Physics, Queen’s University Belfast, Belfast BT7 1NN, United Kingdom; ⊥ School of Chemistry & CRANN Institute & AMBER Centre, Trinity College Dublin, University of Dublin, College Green, Dublin 2, Ireland; # Department of Physics, Florida State University, Tallahassee, Florida 32306, United States

## Abstract

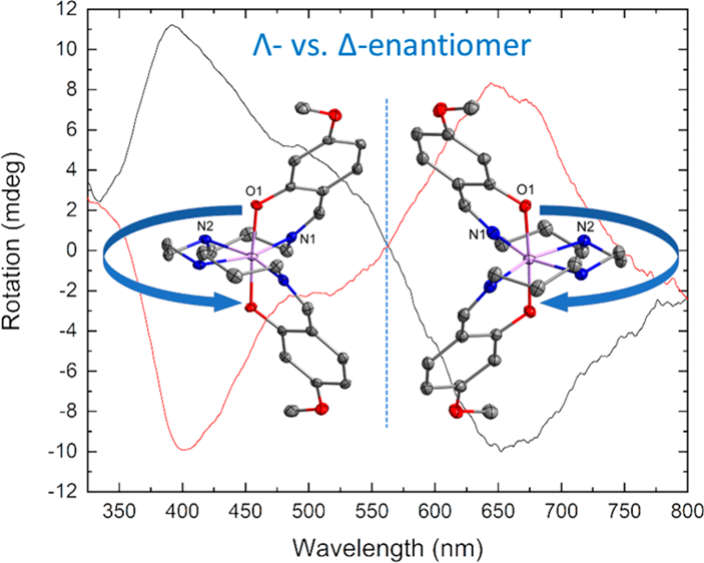

Structural, magnetic, and spectroscopic data on a Mn^3+^ spin-crossover complex with Schiff base ligand 4-OMe-Sal_2_323, isolated in crystal lattices with five different counteranions, are reported. Complexes of [Mn(4-OMe-Sal_2_323)]X where X = ClO_4_^–^ (**1**), BF_4_^–^ (**2**), NO_3_^–^ (**3**), Br^–^ (**4**), and I^–^ (**5**) crystallize isotypically in the chiral orthorhombic space group *P*2_1_2_1_2 with a range of spin state preferences for the [Mn(4-OMe-Sal_2_323)]^+^ complex cation over the temperature range 5–300 K. Complexes **1** and **2** are high-spin, complex **4** undergoes a gradual and complete thermal spin crossover, while complexes **3** and **5** show stepped crossovers with different ratios of spin triplet and quintet forms in the intermediate temperature range. High-field electron paramagnetic resonance was used to measure the zero-field splitting parameters associated with the spin triplet and quintet states at temperatures below 10 K for complexes **4** and **2** with respective values: *D*_*S*__=1_ = +23.38(1) cm^–1^, *E*_*S*__=1_ = +2.79(1) cm^–1^, and *D*_*S*__=2_ = +6.9(3) cm^–1^, with a distribution of *E* parameters for the *S* = 2 state. Solid-state circular dichroism (CD) spectra on high-spin complex **1** at room temperature reveal a 2:1 ratio of enantiomers in the chiral conglomerate, and solution CD measurements on the same sample in methanol show that it is stable toward racemization. Solid-state UV–vis absorption spectra on high-spin complex **1** and mixed *S* = 1/*S* = 2 sample **5** reveal different intensities at higher energies, in line with the different electronic composition. The statistical prevalence of homochiral crystallization of [Mn(4-OMe-Sal_2_323)]^+^ in five lattices with different achiral counterions suggests that the chirality may be directed by the 4-OMe-Sal_2_323 ligand.

## Abstract 2

Zero-field splitting parameters of the spin triplet and quintet forms of a spin-crossover Mn^3+^ complex stabilized in lattices with different counterions are measured by high-field electron paramagnetic resonance at different frequencies. The homochiral crystallization of the enantiopure Δ or Λ forms of the chelate complex, despite the use of achiral anions, is attributed to the steric influence of the ligand substituent.

## Introduction

Manipulation of the internal electronic arrangement in spin-crossover (SCO) complexes,^1−5^ with the attendant changes in magnetic,^6−8^ optical,^9−14^ and electric properties,^7,15−22^ constitutes one of the most versatile ways to build switchable molecular magnets. This versatility is underscored by the varied thermal evolution profiles which characterize spin-state switching. These can range from extremely sharp and hysteretic,^23,24^ particularly suitable for memory applications,^25,26^ to more gradual transitions which have good potential in neuromorphic or sensing roles.^27−29^

**Scheme 1:**
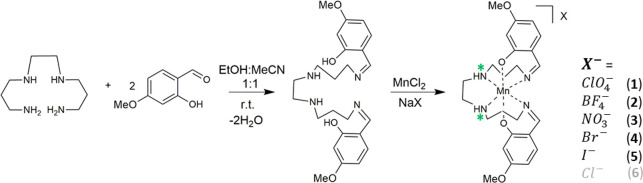
Synthesis Route for Complexes **1**–**6** with the 4-OMe-Sal_2_323 Ligand This ligand type is usually abbreviated as R-Sal_2_323 to indicate the 323 8-carbon alkyl connectivity in the starting tetraamine and the substitution (R) on the phenolate ring. Chirogenic amine nitrogen donors are indicated by an asterisk.

The most studied Mn^3+^ SCO complexes are the mononuclear examples with a hexadentate Schiff base ligand from the R-Sal_2_323 family prepared from condensation of 1,2-bis(3-aminopropyl-amino)ethane with a substituted 2-hydroxybenzaldehyde; for example, see Scheme 1. Metal complexes prepared with hexadentate chelates will be inherently chiral as the Schiff-base ligand has chirogenic amine nitrogen atoms. Crystallization of racemates of the Δ and Λ isomers in centrosymmetric space groups is typical, although recovery of mechanical mixtures of chiral conglomerates of the two forms is also possible but is less common. Here we report the serendipitous crystallization of the SCO complex cation [Mn(4-OMe-Sal_2_323)]^+^ in conglomerate chiral form in five different lattices with achiral counterions. Such homochirality, without use of a chiral anion, has not previously been observed so systematically as is the case for [Mn(4-OMe-Sal_2_323)]^+^ which suggests a ligand-directed effect. In total, [Mn(4-OMe-Sal_2_323)]^+^ was isolated in conglomerate chiral form in lattices with ClO_4_^–^, BF_4_^–^, NO_3_^–^, Br^–^, and I^–^, all crystallizing in spacegroup *P*2_1_2_1_2 and all with a crystallographic *C*_2_ axis bisecting the complex cation. A sixth example, that with a Cl^–^ counterion, was also recovered in space group *Pccn* and the structure only of that complex is included (in the Supporting Information) for the sake of completeness. A check of the CCDC database reveals that only 6 of the 78 unique [Mn(R-Sal_2_323)]^+^ complexes^30^ published before 2021 crystallize adventitiously in a chiral space group,^31−36^ with a seventh example targeted by introduction of a chiral anion.^37^ The interplay between SCO and chirality^38−58^ is increasingly recognized as an important route to switchable nonlinear optical (NLO) materials^51,59−64^ and spin-state dependent changes in optical activity may also constitute an economic and low energy route to follow SCO in sensing applications. Here we use circular dichroism to confirm the spontaneous resolution in the case of the ClO_4_^–^ complex (**1**) and to demonstrate that the complex is stable toward racemization in solution.

**Figure 1:**
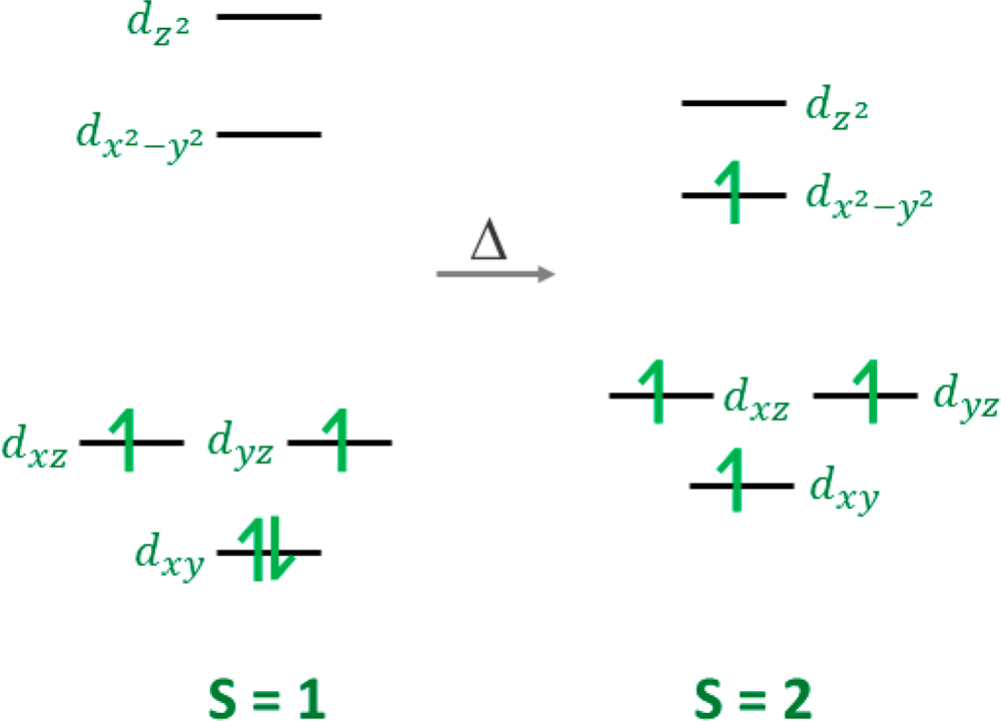
Orbital populations for the spin triplet and axially compressed spin quintet forms of Mn^3+^.

Another less studied aspect of the SCO phenomenon is the associated change in magnetic anisotropy which accompanies the spin pairing/unpairing process and, in particular, the change in magnitude of the zero-field splitting (ZFS) contribution.^65^ The most commonly studied SCO complexes often include the Kramers ions Fe^3+^ and Co^2+^ which have a spin doublet ground state in the fully paired low-spin (LS) configuration and, hence, no ZFS at low temperature, or the non-Kramers Fe^2+^ ion which has a spin singlet ground state when fully paired, i.e., again no ZFS in the cryogenic regime. In contrast, Mn^3+^, which is also a non-Kramers ion and for which thermal SCO is now well-established,^30,31,66−71^ switches between the fully unpaired spin quintet and partially paired spin triplet forms; therefore, a considerable ZFS is expected to persist at low temperature. Spin triplet Mn^3+^ is generally not common, with about 20 examples at room temperature,^32,72−88^ and the ZFS interactions have been quantified via the *D* and *E* parameters in just two cases.^32,83^ These studies have however demonstrated that spin triplet Mn^3+^ has the highest ZFS parameters for any manganese ion, with *D* values in the range +15 to +20 cm^–1^,^65^ while the axially elongated spin quintet form shows small but negative values in the range of −4.5 to −1.2 cm^–1^ with only a small number of published spin quintet Mn^3+^ examples with positive *D* values, i.e., axial compression of the Jahn–Teller ion.^65,89−104^ In these examples, the sign and magnitude of the ZFS parameters have been examined in selected examples by Tregenna-Piggott,^89,90^ Krzystek and Telser,^91,101−103^ and Duboc and Neese,^92,93,99,100^ who have built on the earlier studies of Gregson in probing the electronic structure.^98^ Thermally induced switching to a different spin state in Mn^3+^ can therefore be expected to profoundly affect the magnitude and possibly also the sign of *D*. Identification of the sign of *D* is particularly relevant to the current work because, although the majority of known (HS) Mn^3+^ complexes display a pronounced axial elongation due to the Jahn–Teller effect, most Mn^3+^ SCO complexes appear to have a marked compression in the spin quintet form.^19,31,33,37,69,88,105−112^ An axially compressed^89−100^ form may assist the transition to a spin triplet arrangement as the energetic order of orbitals should match that expected in the *S* = 1 form of the ion (Figure 1). Diffraction alone, however, is not sufficient to discern if this is a genuine compression, but it can be resolved by measuring the sign of *D* by EPR at high fields.

Here we use low-temperature multifrequency EPR spectroscopy to establish the magnitude and sign of the axial *D* parameter in the spin quintet and triplet forms of the [Mn(4-OMe-Sal_2_323]^+^ complex cation^105^ when it is crystallized in BF_4_^–^ and Br^–^ lattices, respectively complexes **2** and **4** in Scheme 1.

In SCO complexes, the geometric structure is heavily dependent on the spin state due to population/depopulation of antibonding orbitals and associated bond length changes. Therefore, we present the temperature-dependent magnetic data at the outset as this guides the choice of temperature for the diffraction studies. The EPR investigation is new and knowledge of the spin state at the temperature of the measurement is essential for the study. Hence, we report EPR measurements and analysis in close alignment with the magnetic results, before discussing structural and optical properties.

## Results and Discussion

### Synthesis

Complexes **1**–**5** were prepared in a one-pot synthesis, Scheme 1, resulting in the formation of dark red/black crystals of the [Mn(4-OMe-sal_2_323)]^+^ compounds (hereafter termed [MnL_1_]^+^) after filtering and standing in air for a few days. [MnL_1_]ClO_4_ (**1**), [MnL_1_]NO_3_ (**3**), and [MnL_1_]Br (**4**) were synthesized directly from the respective Mn(II) salt, while the remaining two complexes were formed by a salt metathesis procedure: Introduction of a tetrafluoroborate or iodide salt led to the formation of [MnL_1_]BF_4_ (**2**) and [MnL_1_]I (**5**), respectively. The structures of all compounds were determined by single-crystal X-ray diffraction before magnetic characterization by SQUID magnetometry and further spectroscopic investigation by high field EPR, UV–vis, and circular dichroism spectroscopies in selected cases (*vide infra*).

Using MnCl_2_·4H_2_O instead of Mn(ClO_4_)_2_·6H_2_O under the same reaction conditions led to the formation of [MnL_1_]Cl·0.34MeOH·3.93H_2_O (**6**), which does not crystallize isomorphously and will therefore not be used further in the magnetic and spectroscopic investigation reported here on **1**–**5**. Synthesis and structural details of **6** can be found in the Supporting Information (section S1).

### Magnetic Characterization of Complexes **1**–**5**

**Figure 2:**
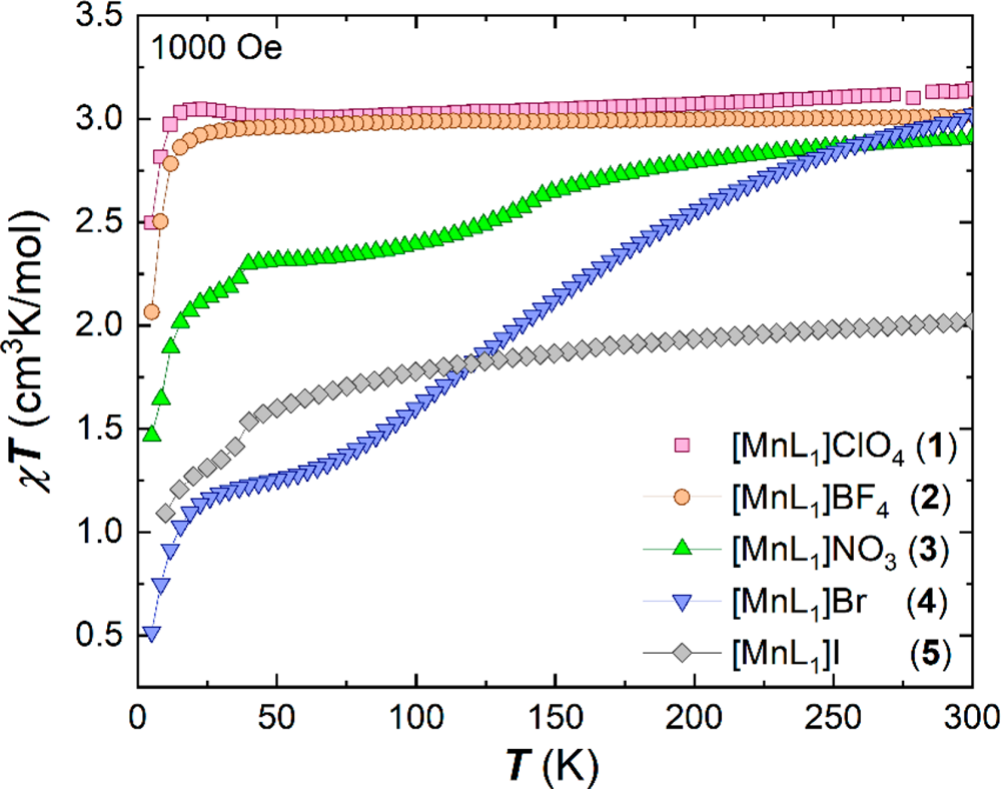
Temperature dependence of the χ_M_*T* products at 1000 Oe in cooling mode for all five complexes **1**–**5**.

The magnetic susceptibility, χ_M_, of the bulk samples of compounds **1**–**5** was measured using a SQUID magnetometer on cooling from 300 to 5.0 K under an applied direct current (dc) field of 1000 Oe, shown as temperature dependence in the form of the χ_M_*T* product (Figure 2).

Complexes **1** and **2** are high-spin over the measured temperature range with values close to the expected spin only value of 3.0 cm^3^ K/mol for a monomeric Mn^3+^ complex with *S* = 2 and *g* = 2. The room temperature value of 2.9 cm^3^ K/mol for complex **3** indicates that the full high-spin *S* = 2 state is not reached at room temperature and, on cooling, the χ_M_*T* value decreases steadily in a two-step transition, which becomes clearer in the derivative of the χ_M_*T* product (see Figure S1), where maxima at 142 and 37 K are apparent. The χ_M_*T* value displays a plateau at 2.3 cm^3^ K/mol between these two temperature points, which is indicative of a 2:1 ratio of HS/LS sites. In order to confirm the second step at lower temperatures, the susceptibility was additionally measured in warming mode (see Figure S1). Bromide complex **4** is the only compound that exhibits a full thermal spin transition from *S* = 2 to *S* = 1 (expected spin-only value of 1.0 cm^3^ K/mol using *S* = 1 and *g* = 2) between 300 and 70 K, following a gentle sigmoidal pathway and with *T*_1/2_ of 136 K. The χ_M_*T* value of 2.0 cm^3^ K/mol at room temperature for complex **5**, with the larger iodide counterion, indicates a likely 1:1 mixture between the spin triplet and quintet sites within the crystal lattice. The χ_M_*T* product remains almost constant over the measured temperature range upon cooling, before a decrease below 35 K, suggesting a further adjustment to the spin-state ratio, as this temperature is too high for zero-field splitting effects, which are typically observed below 25 K.

### High-Field Electron Paramagnetic Resonance Spectra of **2** and **4**

**Figure 3:**
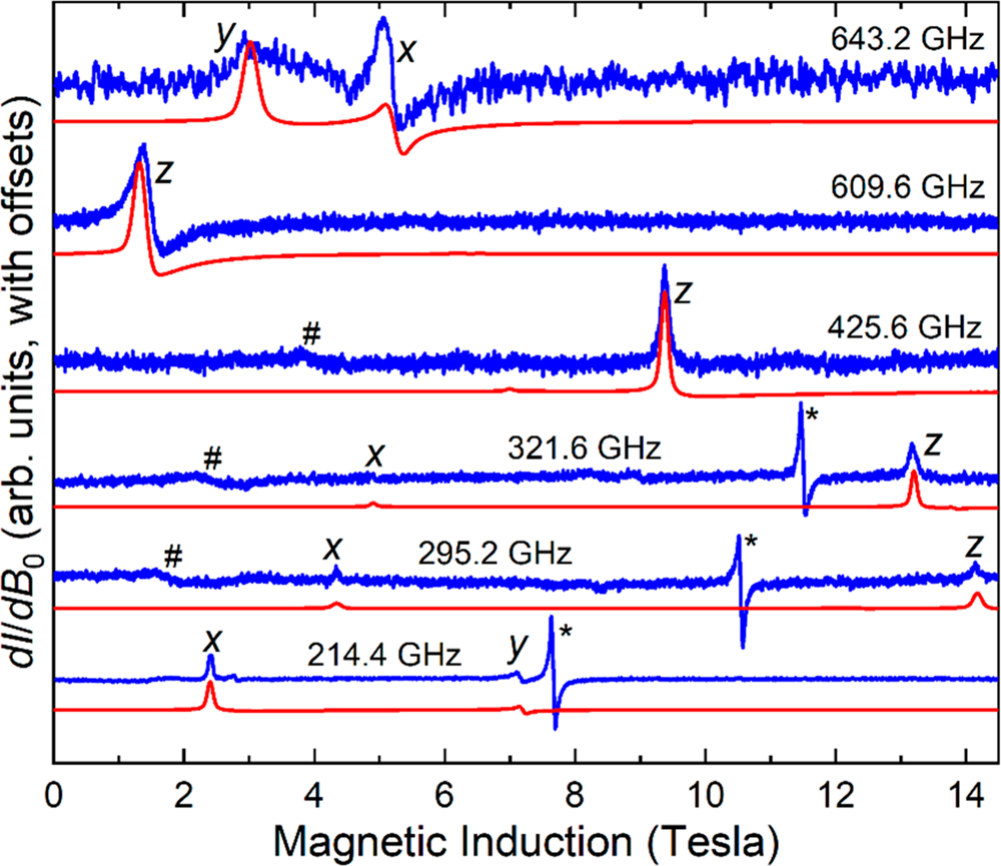
Derivative mode (d*I*/d*B*_0_, where *I* is the microwave intensity transmitted through the sample and *B*_0_ the applied magnetic field) HFEPR spectra of **4** recorded at 10 K and frequencies as indicated. The experimental spectra are shown in blue and simulations in red, generated using the parameters given in the main text. The resonances are labeled according to the associated components (*x*, *y*, and *z*) of the powder spectrum. The sharp feature at the *g* = 2.00 position, marked with an asterisk (*), is attributed to a Mn^2+^ impurity; # denotes a weak signal possibly due to a small *S* = 2 Mn^3+^ contaminant.

High-field EPR (HFEPR) spectra were recorded at low temperatures (∼10 K) on polycrystalline powder samples of compounds **2** and **4** in order to characterize the ZFS parameters associated with the HS and LS species, respectively. We first present the results for compound **4**, which undergoes a complete transition to the LS state below ∼50 K, resulting in very clean and simple HFEPR spectra, some of which are displayed in Figure 3.

**Figure 4:**
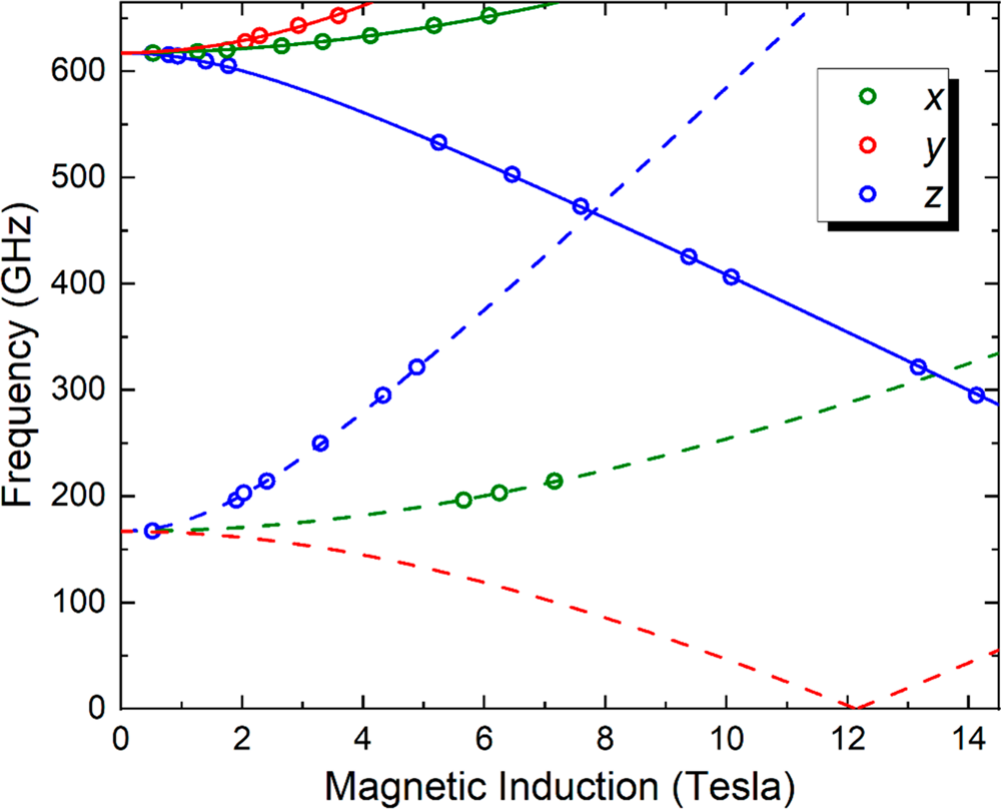
2D frequency versus resonance field plot for compound **4** at 10 K. The circles denote experimental data points, and the curves are fits to an effective spin *S* = 1 Hamiltonian [eq 1] with the parameters given in the main text. Colors denote different components (*x*, *y* and *z*; see legend) of the powder spectra; the solid curves indicate cold transitions (assuming *D* > 0) originating from the ground state of the *S* = 1 manifold and, therefore, persist to the lowest temperatures. The dashed curves indicate transitions within excited states of the *S* = 1 manifold.

Resonance peak positions determined from HFEPR spectra for **4** such as those in Figure 3 (and several others not shown) were then used to construct a 2D frequency versus field map, as shown in Figure 4, with colors denoting turning points due to the three principal (*x*, *y*, and *z*) components of the powder-averaged spectra. These data were then fit to the following spin Hamiltonian:^113^
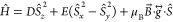
1assuming a spin *S* = 1 ground state. The first and second terms in eq 1 respectively denote the axial and rhombic second-order ZFS interactions, with the associated *D* and *E* parameters. Meanwhile, the last term parametrizes the Zeeman interaction in terms of an anisotropic -tensor. *Ŝ* is the total spin operator with components *Ŝ*_*i*_ (*i* = *x*, *y*, and *z*),  is the magnetic induction, and μ_B_ is the Bohr magneton. The data indicate more than one zero-field energy gap,^114^ requiring a finite, albeit relatively small rhombicity factor *E*/*D* = 0.117 (the lowest frequency intercept corresponds exactly to 2*E* = 167 GHz = 5.58 cm^–1^).^115^ The spectral simulations in Figure 3 were then generated with the following parameters: *g*_*x*_ = 1.97(2), *g*_*y*_ = 2.13(5), *g*_*z*_ = 2.00(1), *D* = +23.38(1) cm^–1^, and *E* = +2.79(1) cm^–1^. The relative intensities of the *z* and *x* spectral peaks confirm the positive sign of the axial ZFS (*D*) parameter. These parameters were used to simulate the low-temperature dc magnetic susceptibility of **4** (Figure S2). We note that the obtained *D* and *E* values are slightly larger than those reported previously for a similar LS Mn^3+^ compound, [Mn(napsal_2_323)]NTf_2_.^32^

Finding one *g*-tensor component that is larger than 2.00 for a d^4^ electronic configuration is initially surprising, as one normally anticipates values lower than 2.00 for a less than half-filled d-shell. However, the non-Hund’s rule LS configuration may be reduced to that of a greater than half-filled *t*_2g_ set for an octahedral coordination, i.e., 4 electrons (2 holes) occupying the three *t*_2g_ orbitals.^116^ Distortions away from octahedral geometry may then give *g*-tensor components greater than 2.00.

**Figure 5:**
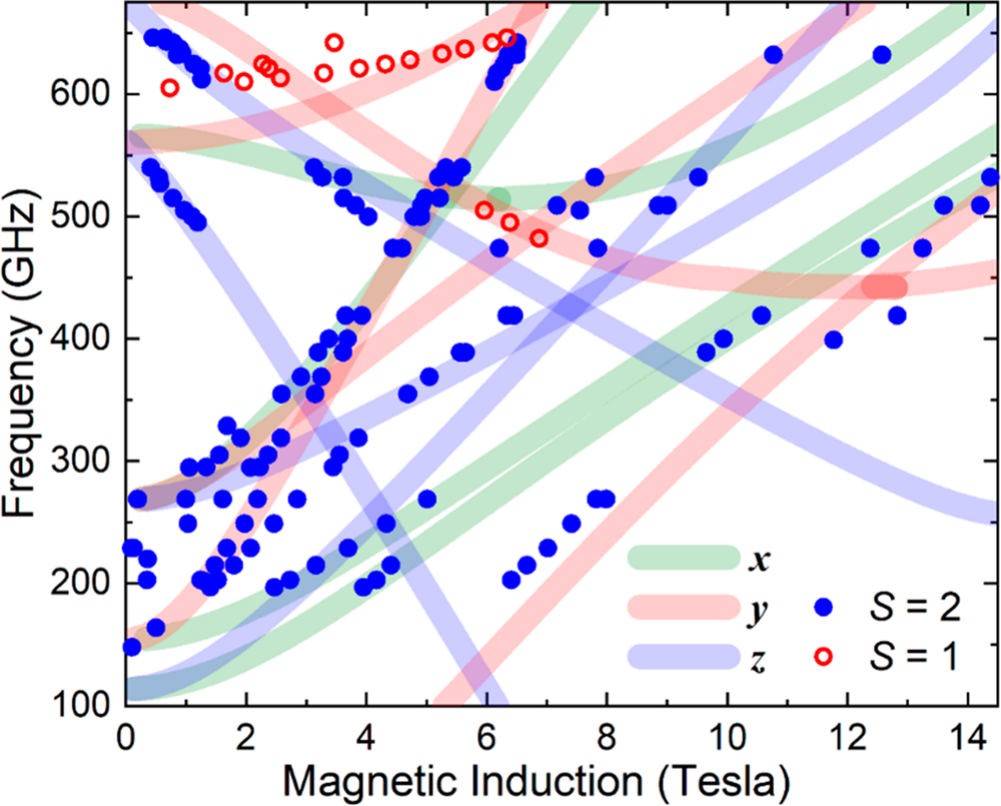
2D frequency versus resonance field plot for compound **2** at 5 K. The circles denote experimental data points [closed (blue) and open (red) symbols indicate suspected *S* = 2 and 1 features, respectively], and the thick curves represent the best simulation (colors denote *x*, *y*, and *z* components; see legend) based on an effective spin *S* = 2 Hamiltonian [eq 1] with the parameters discussed in the main text. Thick lines are employed to note the much larger uncertainty in the ZFS parameters in comparison to compound **4**.

Compound **2** was selected because magnetic measurements (Figure 2) suggest that it remains in the *S* = 2 HS state down to the low temperatures necessary for achieving sufficient HFEPR sensitivity. The resultant powder spectra turned out to be surprisingly difficult to interpret. First, the increased spin multiplicity inevitably results in many more spectral features, especially when considering both parallel and perpendicular mode transitions and off-axis peaks.^117^ The 2D frequency versus resonance field maps generated from multifrequency measurements collected at 5 K are displayed in Figures 5 and S3–S5. Attempts to simulate the results start by following the evolution of signals to zero field, thus gaining information on the ZFS without dependence on the *g*-tensor. In the present case, there are very obvious high-frequency intercepts at ∼560 and ∼675 GHz that fit well with the *S* = 2 model. However, there is another series of weaker peaks (red open circles) with an intercept at ∼600 GHz that is incompatible with an *S* = 2 state, which appears to better fit to the *S* = 1 parametrization in Figure 4 (with a slightly lower intercept value). These resonances can be fit assuming *S* = 1 and *g*_*x*_ = 2.03(6), *g*_*y*_ = 2.16(2), *g*_*z*_ = 2.0(2), *D* = +23.3(8) cm^–1^, and *E* = +3.1(8) cm^–1^; they are, thus, not very different from the ZFS parameters of **4** and also exhibit the same *g*_*y*_ > 2 issue. The parameter errors are large due to the relatively small number of available data points and low signal quality. Therefore, we suspect that either some sites in crystals of **2** convert to a LS state or that the powder sample is contaminated with a small fraction of microcrystals that undergo a transition to a LS state, perhaps caused by stresses induced when grinding the sample. These findings illustrate the value in carrying out HFEPR in order to deduce ZFS parameters, as the mixtures of spin states would render any parameters deduced from a purely thermodynamic measurement highly unreliable.

**Figure 6:**
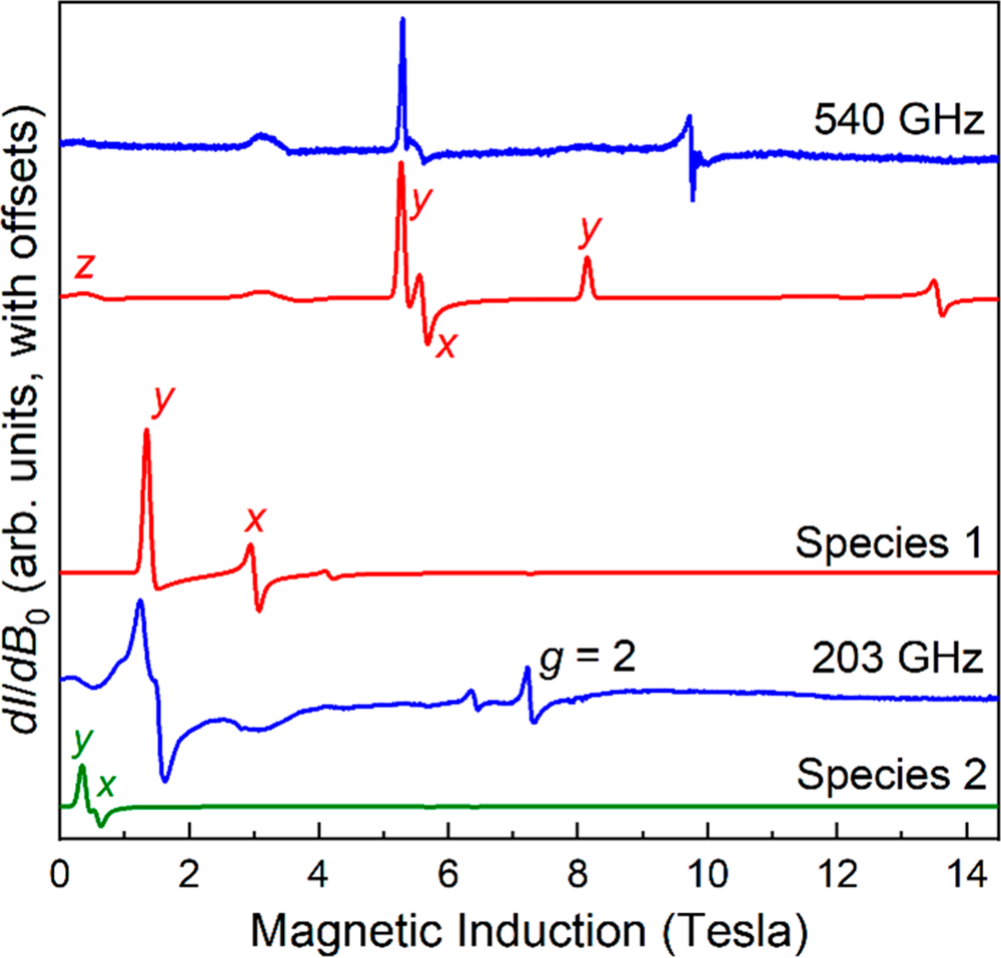
Derivative mode HFEPR spectrum of **2** recorded at 5 K and frequencies indicated. The simulations in red were generated at 540 and 203 GHz using *g*_*x*_ = 1.972, *g*_*y*_ = 1.978, *g*_*z*_ = 1.94, *D* = *+*6.87 cm^–1^, and *E* = +0.63 cm^–1^. The 203 GHz simulation in green used *g*_*x*_ = 1.972, *g*_*y*_ = 1.978, *g*_*z*_ = 1.94, *D* = +7.17 cm^–1^, and *E* = +0.17 cm^–1^. The resonances are labeled according to the associated components (*x*, *y* ands *z*) of the powder spectrum. The sharp feature at the *g* = 2.00 position in the 203 GHz spectrum is attributed to a Mn^2+^ impurity.

After constraining the high-frequency intercepts, it is found that the best *S* = 2 simulation [based on eq 1] results in additional zero-field intercepts at lower frequencies that fall in a range (150–275 GHz) where many low-field peaks are observed. However, no single parametrization reproduces all peak positions. We therefore believe that the powder sample also contains multiple *S* = 2 species with a small spread in ZFS parameters;^118^ to reflect this, we employ broader/lighter simulated curves in Figure 5. The existence of different species may be due to distinct sites within the lattice of an individual crystal,^119^ or they could be due to crystal-to-crystal variations within the powder. A conservative analysis indicates the following axial ZFS parameter, *D* = +6.9(3) cm^–1^, and a much more significant spread in *E*, with values from 0.17 to 0.63 cm^–1^ needed to reproduce all observed resonances (see Figures 5 and S3–S5). Again, the sign of *D* is constrained via the relative intensities of the different peaks. Indeed, a high-frequency spectral simulation that assumes *D* = +6.9(3) cm^–1^, *E* = +0.63 cm^–1^, *g*_*x*_ = 1.97, *g*_*y*_ = 1.98, and *g*_*z*_ = 1.94 is in excellent agreement with the corresponding experimental spectrum, as displayed in Figure 6. The frequency versus field plots calculated with the two sets of parameters are shown in Figures S3–S5.

Finally, we comment on a possible relationship between the *D* parameters obtained for the HS and LS states. Here, we have assumed that the main contribution to the ZFS comes from spin–orbit coupling (SOC). It then follows that the Ligand-Field theory perturbative expression for *D* consists of three terms: (1) a numerator proportional to the SOC constant, ζ, and the sum of the squared matrix elements of the *L̂*_*z*_ operator between the ground and excited orbital states; (2) a denominator corresponding to the associated orbital excitation energies; and (3) a prefactor, 1/*S*^2^.^120^ The first two terms may not vary significantly between the HS and LS states, as they primarily involve excitations between essentially the same orbital states in either (octahedral) case. However, the prefactor obviously differs by a factor of 4 between LS and HS states, potentially explaining the majority of the reduction in *D* between the two configurations (all other things being equal), i.e., *D*_*S*__=1_/*D*_*S*__=2_ = 3.4. Interestingly, for a pure *DŜ*_*z*_^2^ description of the ZFS Hamiltonian, the largest zero-field gap in the spectrum is proportional to (2*S* – 1) [= *S*^2^ – (*S* – 1)^2^] and should, thus, differ by a factor of 3 for the *S* = 1 and 2 states with identical *D* values. The fact that *D*_*S*__=1_/*D*_*S*__=2_ ≈ 3 in the present case explains why the zero-field intercepts are close to 600 GHz for both states in Figures 4 and 5, i.e., the different *D* values and (2*S* – 1) factors more or less cancel, leading to similar aggregate magnetic anisotropies (as measured by the ZFS) for the two compounds.

### Theoretical Calculations

Zero-field splitting is a result of spin–orbit coupling and ligand field splitting of energy levels of a paramagnetic atom possessing spin larger than 1/2. Dependencies between the ZFS and the ligand field energies for various electronic configurations are well-known,^121−125^ but they are often difficult to apply as the ligand field bands are obscured by the charge-transfer bands. Calculations of the ZFS parameters for the Mn^3+^ ions were thus attempted using the state-averaged complete active space self-consistent field (CASSCF) method as implemented in the ORCA 5.0.1 quantum mechanical software package.^126−129^ Four electrons in five orbitals were used in the calculations; five quintets and the lowest ten triplet and ten singlet states were taken into account.^129−131^ The initial quasi-restricted orbitals (qro) were obtained from a DFT calculation employing the B3LYP/G functional and the diffuse def2-TZVPP function basis set for all atoms.^132^ The Ahlrich’s auxiliary basis sets were embedded into the ORCA software.^133,134^ In the case of complex **2** (BF_4_^–^), the calculation produced *D* = +3.84 cm^–1^, compared to the experimental *D* = +6.9(3) cm^–1^. The calculated *E*/*D* ratio of 0.11 compares reasonably with the experimental values that range from 0.025 to 0.09. The largest contribution to *D* (2.209 cm^–1^) comes from the lowest triplet state, derived from the free-ion term ^3^H. In contrast, calculations of *D* in the *S* = 1 state of complex **4** (Br^–^) were less satisfactory, and despite using the wave functions from an *S* = 1 DFT calculation, the CASSCF procedure converged to the S = 2 state. Using the structures optimized by the ORCA DFT calculations (Table S1) did not result in an improvement (see section S2 of the Supporting Information).

### Structural Characterization of Compounds **1**–**5**

**Figure 7:**
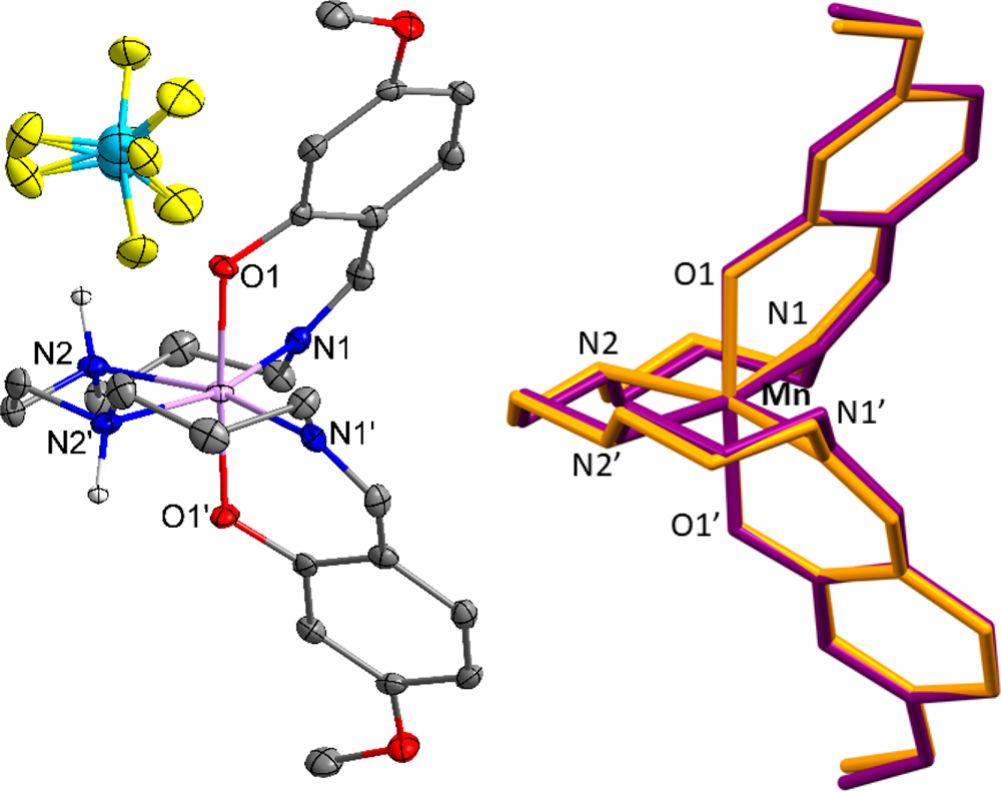
Molecular structure of complex **2**, [MnL_1_]BF_4_ (hydrogen atoms omitted for clarity) (left), and structural overlap of the cationic species of complex **2**, [MnL_1_]BF_4_ (yellow) and complex **4**, [MnL_1_]Br (purple) at 100 K (right).

All complexes **1**–**5** crystallize isostructurally in the orthorhombic space group *P*2_1_2_1_2, with *Z* = 2, where the asymmetric unit contains half of a [Mn(4-methoxy-sal_2_323)]^+^ cation, as shown in Figure 7, and half of the respective anion, both located on a symmetry center. All complexes crystallized solvent-free.

The coordination around the Mn^3+^ center is pseudo-octahedral with two *trans*-phenolate, two *cis*-amine, and two *cis*-imine donors, in the same arrangement as reported for related [Mn(R-sal_2_323)]^+^ SCO compounds.^19,30,31,33,88,105−107,135^ Compounds **1**–**5** provide a good set of samples to study the effect of the counterion on the Mn^3+^ spin state, since all five compounds crystallize isotypically.

Typically the average bond lengths in [Mn(R-sal_2_323)]^+^ type complexes change upon spin transition, but only significantly for the amine and imine bonds in the equatorial positions. Reported bond length changes for Mn–N_imine_ donors are of the order of 1.9–2.0 Å (*S* = 1) to 2.1–2.2 Å (*S* = 2), while those for the Mn–N_amine_ bond lengths are 2.0–2.1 Å (*S* = 1) to 2.2–2.3 Å (*S* = 2).^110^ The bond lengths of complexes **1**–**5** are summarized in Table 1 and clearly show the impact of the anion in the crystal lattice on the spin state of the Mn^3+^ cation. At 100 K, compounds **1** and **2**, with higher volume tetrahedral anions, have bond lengths typical for an *S* = 2 species, while those for complex **4** with the Br^–^ counterion are in the correct range for *S* = 1, in line with the SQUID data. At 100 K, bond lengths for nitrate and iodide complexes **3** and **5** suggest a mixture of spin triplet and quintet states, again in line with the magnetic data.

**Table 1:** Mn^3+^ Bond Lengths in Different Spin States of **1**–**5**

Mn–X	ClO_4_^–^ (**1**)	BF_4_^–^ (**2**)	NO_3_^–^ (**3**)	Br^–^ (**4**)	I^–^ (**5**)
temp (K)	100	100	100	100	100
Mn–O_phen_	1.878	1.881	1.890	1.899	1.887
Mn–N_imine_	2.102	2.098	2.060	2.012	2.073
Mn–N_amine_	2.227	2.221	2.165	2.085	2.178
spin state	*S* = 2	*S* = 2	mixed	*S* = 1	mixed
temp (K)	a	a	293	190	a
Mn–O_phen_			1.884	1.892	
Mn–N_imine_			2.092	2.051	
Mn–N_amine_			2.197	2.139	
spin state			*S* = 2	mixed	

a Not measured as magnetism not temperature dependent above 100 K.

Upon warming, the bond lengths of complex **3** show the expected equatorial elongation (see Table 1) and indicate the transition to a state with a higher percentage of the *S* = 2 species. Structural data were also collected at higher temperature (190 K) for complex **4**, [MnL_1_]Br, revealing the bond lengths to be similar to those of complex **3**, [MnL_1_]NO_3_, at 100 K. This is in good agreement with the magnetic data for complex **4**, [MnL_1_]Br, where the χ_M_*T* value at 190 K is 2.48 cm^3^ K/mol, i.e., a value similar to that for complex **3** at 100 K, which shows an almost identical value of 2.40 cm^3^ K/mol, indicating a 2:1 ratio of quintet:triplet sites. Comparison of the Mn–nitrogen (amine or imine) bond lengths in the pure *S* = 1 state, for example, those of complex **4** at 100 K, with equivalent donors in the pure *S* = 2 state, for example, those of complex **1** at 100 K (Table 1), provide a useful scale of the bond length difference equivalent to 100% spin conversion. Therefore, distances within that range can be used to make a good estimate of the relative percentage of the two spin states at any temperature. For example, SQUID data for complexes **3** and **4** at 100 and 190 K, respectively, indicate a 2:1 ratio of quintet/triplet states (Figure 2). This is in good agreement with the closeness of the Mn–nitrogen bond lengths for these complexes at the indicated temperatures (Table 1). In the case of complex **3**, the stepped profile of the SCO suggests an ordered phase at 100 K, whereas the gradual profile in complex **4** suggests a Boltzmann distribution of spin states at 190 K.

Since compounds **1**–**5** crystallize isotypically in the orthorhombic space group *P*2_1_2_1_2, the difference in spin state must be due to packing and intermolecular interactions. In all cases the [MnL_1_]^+^ cations arrange in a parallel way forming 1D chains along the *b*-axis (see Figure S7). These chains pack into a zigzag formation due to the crystallographic symmetry elements of the orthorhombic *P*2_1_2_1_2 space group. We suggest that the consistent recovery of enantiopure individual crystals of either the Λ or Δ forms of the associated manganese complex with a range of achiral counterions may be due to the unique position and size of the ligand substituent in the 4-OMe-Sal_2_323 ligand, i.e., a methoxy group para to the phenolate donor. We also suggest that steric effects of this substituent arrangement disfavor packing of both the Λ and Δ enantiomers of the complex cation in the presence of the medium sized counterions reported here: ClO_4_^–^, BF_4_^–^, NO_3_^–^, Br^–^, and I^–^. With smaller counterions, including Cl^–^ (compound **6**, structure reported in the Supporting Information), or larger counterions including CF_3_SO_3_^–^, PF_6_^–^, and BPh_4_^–^,^105^ the packing pattern is altered, and the crystallization of both enantiomers is observed, as is typical for this type of chiral complex with achiral anions.

Compounds **1** and **2** with tetrahedral perchlorate and tetrafluoroborate anions have closely related intermolecular interactions, with H-bonding between one anion and four neighboring cations leading to a 3D network (see Figure S8). A similar behavior is observed in the room-temperature structure of complex **3**, [MnL_1_]NO_3_ (see Figure S9). Upon cooling, the packing arrangement shows a weakening of the H-bonds, but each anion still exhibits short contacts to four neighboring cations. Complex **4**, [MnL_1_]Br, exhibits intermolecular H–Br bonds at 100 K, where one bromide anion shows close contacts to three neighboring cations (see Figure S10), which increases to four neighboring cations upon warming to 190 K. Complex **5**, containing the slightly bigger iodide anion, exhibits short contacts at 100 K to four neighboring cations (see Figure S11), in a fashion similar to that observed for compounds **1** and **2** and the room-temperature structure of complex **3**.

Spin-crossover Mn^3+^ compounds exhibit a stronger distortion of the octahedral environment in the *S* = 2 state than in the almost perfect octahedron associated with the *S* = 1 state due to loss of the Jahn–Teller distortion upon cooling, as the antibonding orbital is depopulated. The degree of distortion can be analyzed by the distortion parameters Σ and Θ, as defined by McKee et al.,^136^ where Σ highlights the angular deviation from the 90° *cis*-octahedral angles, and Θ measures the trigonal distortion from a perfect octahedral environment toward trigonal prismatic geometry. In the case of a perfect octahedron, both values are zero. The reported literature values for typical spin-crossover Mn^3+^ compounds are Σ = 28–45° for *S* = 1 (Σ = 48–80° for *S* = 2) and Θ = 79–125° for *S* = 1 (Θ = 135–230° for *S* = 2)^19^ and these values can be used to help assign the spin state.

Σ and Θ have been calculated for **1**–**5** using OctaDist 2.6.1,^137^ and the observed parameters (Table 2) are in line with the assigned spin states. Some anomalies include the high trigonal torsion parameter Θ for the spin triplet form of **4**, [MnL_1_]Br, but the angular distortion is in line with other *S* = 1 complexes. Upon warming, the values for complex **3**, [MnL_1_]NO_3_, increase slightly to Σ = 58.42° and Θ = 221.46°, highlighting the full conversion to the high-spin state. In the case of complex **4**, [MnL_1_]Br, the first temperature increase to 190 K reveals that the distortion parameters change to Σ = 49.73° and Θ = 179.76°, reflecting the gradual spin-state change on warming. An overlay of the complex cations of compounds **2** and **4** in spin quintet and triplet forms, respectively, highlights the differences in local distortion (Figure 7, right). While most of the Schiff base ligand overlaps almost perfectly, there are small discrepancies visible in the amine backbone as well as the peripheral methoxy substituent.

**Table 2:** Distortion Angle Parameters Σ (Angular Deviation at the origin) and Θ (Trigonal Torsion Angle) for [MnL_1_]X Complexes **1**–**5** at 100 K

	ClO_4_^–^ (**1**)	BF_4_^–^ (**2**)	NO_3_^–^ (**3**)	Br^–^ (**4**)	I^–^ (**5**)
Σ	61.67	59.61	53.21	39.12	57.43
Θ	233.83	225.89	202.41	141.41	214.87
spin state	*S* = 2	*S* = 2	mixed	*S* = 1	mixed

### CD and UV–Vis Spectroscopy

**Figure 8:**
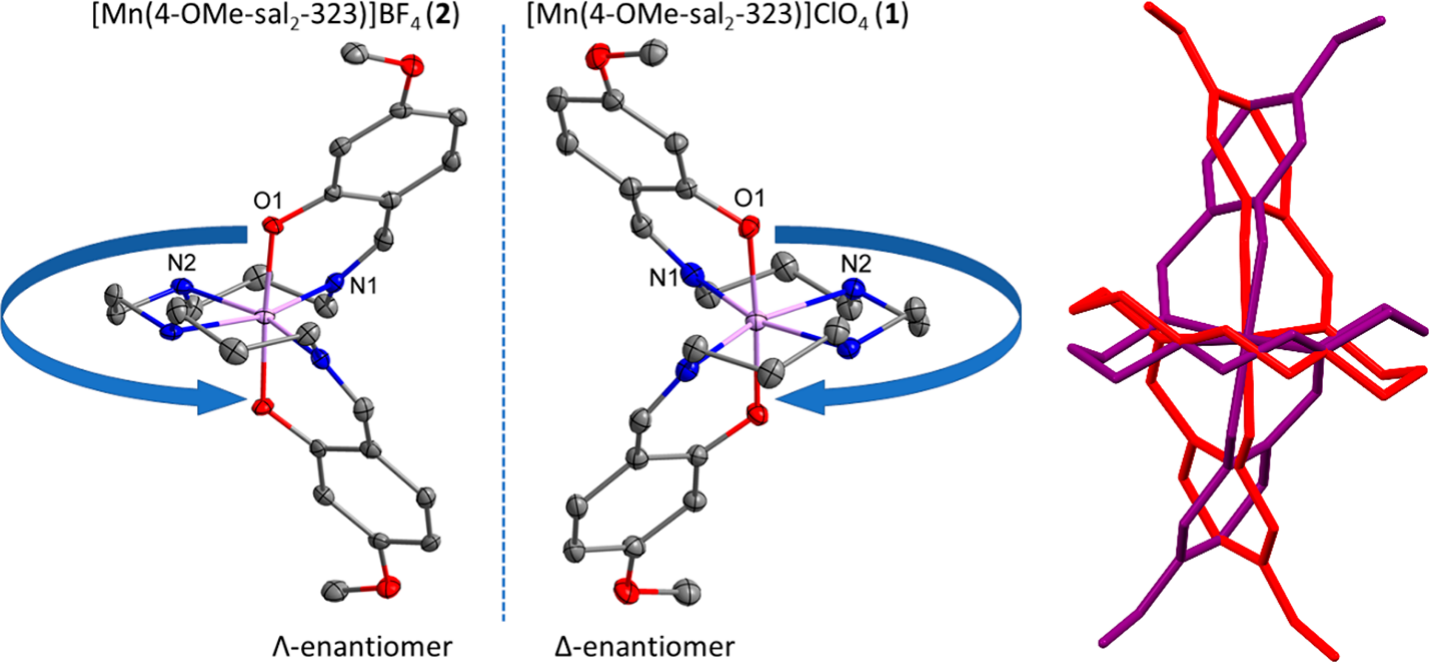
Left: Structures of **2**-Λ (left) and **1**-Δ (right) shown as representative enantiomers. Right: Overlay of enantiomers of bromide complex **4**-Λ and **4**-Δ, collected on different crystals at different temperatures, where the O–Mn–O axes from both are aligned (hydrogen atoms and counterions omitted for clarity).

During the X-ray structure collection and analysis of compounds **1**–**5** at various temperatures, each single crystal was internally enantiopure, but both enantiomers have been observed (Table 3). The appearance of both the dextro, Δ, and laevo, Λ, enantiomers highlights that the bulk material consists of a conglomerate of both enantiomers. Within the seven structures determined over different temperatures, we have observed a close-to 50:50 ratio distribution of the two enantiomers Λ/Δ, as highlighted in Figure 8 and Table 3.

**Table 3:** Enantiomer Determination from Single Crystal Structural analysis on Seven Different Crystals

	ClO_4_^–^ (**1**)	BF_4_^–^ (**2**)	NO_3_^–^ (**3**)	Br^–^ (**4**)	I^–^ (**5**)
100 K	Δ	Λ	Δ	Λ	Δ
*T* > 100 K			Λ	Δ	

**Figure 9:**
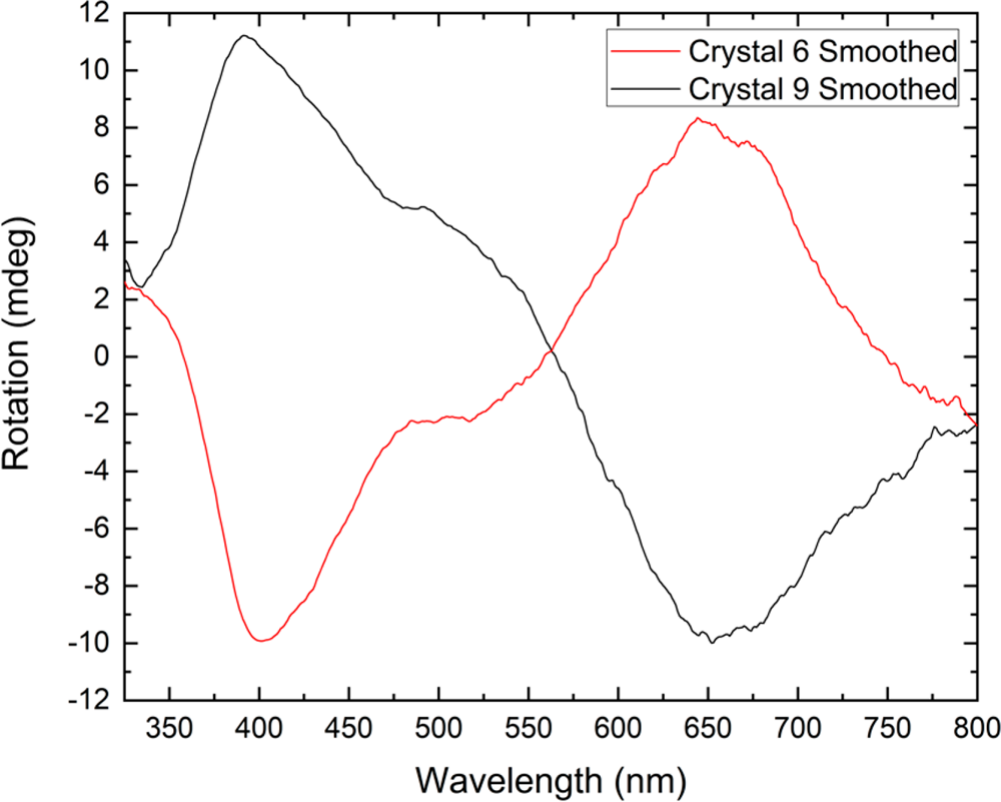
Solution circular dichroism spectroscopy on two dissolved crystals of **1** each dissolved separately in methanol indicating the two enantiomers. Smoothing applied for the eye; unsmoothed graph available in the Supporting Information.

Given the chiral nature of the complex and the crystallization in a chiral space group, circular dichroism (CD) spectroscopy was recorded at room temperature. Initial attempts to directly measure individual crystals of all compounds in the solid state showed optical rotation, although the resolution was poor. A solution-state study was then completed on high-spin compound **1** for which there was the highest yield of single crystals and which therefore offered the best chance of statistical analysis. The enantiomeric forms were clearly distinguishable on measurements of methanol solutions of individual single crystals of **1** (Figure 9), indicating they do not racemize in solution. Statistical studies in methanol solution on three batches each of 10 single crystals indicate an approximate ratio of 2:1 of the two enantiomers within each batch, with an overall distribution of 20:10 from 30 investigated crystals. All the solution spectra are included in Figures S12–S14.

Solid-state UV–vis spectra were collected for complexes **1** and **5** at room temperature, i.e., in a temperature regime where [MnL_1_]^+^ is fully in the *S* = 2 form (complex **1**) and stabilized with a mixture of *S* = 1 and *S* = 2 forms (complex **5**). The spectra for both have strong features in the 200–400 nm range (Figure S15). In addition, a broad but weak shoulder around 525 nm was observed for both compounds (Figure S15). The intensity ratio of the peaks at 225, 255, and 305 nm is different for the pure *S* = 2 sample (**1**) and the mixed *S* = 1/*S* = 2 sample, (**5**), suggesting that the more intense feature at 225 nm is associated with the *S* = 1 electronic state (Figure S15).

Solution UV–vis spectroscopy of the free ligand H_2_L_1_ (Figure S16) in acetonitrile confirms the origin of the peaks at 225, 255, and 305 nm as being ligand-based and demonstrates that coordination to the metal ion in different spin states can result in different ligand-to-metal charge transfer (LMCT) bands in the solid state (Figure S15). Although the solution spectra of complexes **1** and **5** were recorded in methanol (Figure S17), a meaningful comparison with the spin states is not possible as the SQUID data were recorded only in the solid state. However, some differences were detected, notably a change in intensity of the UV absorption at 250 nm and a new absorption at around 770 nm for iodide complex **5** (Figure S17).

## Conclusions

Isolation of a cationic Mn^3+^ complex in lattices with five different counterions resulted in stabilization of the ion in either the *S* = 2 state (complexes **1** and **2**) or promoted thermal spin crossover behavior (complexes **3**–**5**). HFEPR was used to estimate the magnitude and sign of the axial *D* parameter in both spin states by recording low-temperature variable-frequency spectra on complexes **2** and **4**. This confirmed that the spin quintet form is axially compressed with a *D* value of +6.9(3) cm^–1^ which increased to *D* = +23.38(1) cm^–1^ in the spin triplet form. Both spin triplet and axially compressed spin quintet electronic states are less common for Mn^3+^ complexes, and the results here are in line with the small number of published examples of each type. This study has demonstrated that HFEPR is an effective method to follow thermal spin transitions in Mn^3+^ and may also have potential as a probe for nonthermal switching, for example by application of light or a magnetic field. Serendipitous crystallization of complexes **1**–**5** in the space group *P*2_1_2_1_2 highlights the inherently chiral nature of Mn^3+^ complexes with the R-Sal_2_323 ligand type and how this feature may have potential to be coupled to changes in spin state. Use of circular dichroism spectroscopy enabled a statistical analysis of separate solutions of each of 30 single crystals of high-spin complex **1** which revealed a 2:1 weighting of Λ and Δ enantiomers in this sample, and solution measurements on the same compound show that the complex does not racemize over a few days. The spontaneous homochiral crystallization of [Mn(4-OMe-Sal_2_323)]^+^ with different achiral counterions suggests a ligand-directed effect which we have not previously observed. In contrast the choice of counterion has a more direct effect on the choice of spin state within the isotypical homochiral series, with larger counterions (ClO_4_^–^ and BF_4_^–^) stabilizing the spin quintet form, while smaller ones (NO_3_^–^ and Br^–^) tend to promote SCO. Our studies on related systems continue on both chiral and nonchiral examples.

## Experimental Section

### General Experimental Details

#### Physical Measurements

All measurements were recorded on powdered samples of the respective polycrystalline compound. Elemental analyses (C, H, and N) were carried out using a PerkinElmer Vario EL instrument and mass spectra were recorded on a Waters 2695 Separations Module Electrospray Spectrometer. Solution and solid-state UV–vis spectra were recorded on an Agilent Cary 60 UV–vis spectrometer. Solution measurements were carried out on ∼1.5 × 10^–5^ M methanolic solutions of samples **1** and **5**. Solid-state samples of complexes **1**, **2** and **5** were prepared for UV–vis by grinding a small number of crystals with a few drops of silicone oil. The resulting paste was thinly spread on the edge of a quartz cuvette for collection.

CD spectra were recorded on a JASCO J-810 spectrometer. Solutions were prepared by systematically taking a single crystal of **1** and dissolving it in methanol (1 mL). The process was repeated 30 times to acquire a large distribution of samples. Solid-state samples were prepared by grinding a single crystal of **1** with 50 mg of KBr which was pressed into a disk under 12.5 tonnes of pressure for 90 s. This was then pasted onto the side of a cuvette using silicone oil, and the CD of the solid-state disc was recorded. It is important to note that a variety of single crystals with different size and shape were chosen. All CD spectra were recorded with a scan rate of 100 nm/min. Powder X-ray diffraction (PXRD) experiments were carried out for complex **3**, [MnL_1_]NO_3_, using a Bruker D2 Phaser with Cu Kα radiation (λ = 1.5418 Å). The powder diffraction pattern of complex **3** is shown in section S3 of the Supporting Information.

### Magnetic Measurements

The magnetic susceptibility measurements were recorded on a Quantum Design SQUID magnetometer (MPMS-XL) operating between 1.8 and 300 K. Direct current measurements were carried out on polycrystalline samples prepared in gelatin capsules in a field of 0.1 T. Diamagnetic corrections were applied to correct for a contribution from the sample holder, and the inherent diamagnetism of the sample was estimated with the use of Pascal’s constants.

### High-Field EPR Spectroscopy

HFEPR spectra were recorded at the National High Magnetic Field Laboratory (NHMFL, Tallahassee, FL) using the homodyne transmission spectrometer equipped with a 15/17 T superconducting magnet.^138^ Measurements were carried out on powder samples of **2** and **4** (∼30 mg) mixed with eicosane wax. The samples were packed into a Teflon sample holder. Spectra were recorded at different temperatures and multiple frequencies from 203 to 634 GHz in the 0 to 14.5 T field range.

### Materials and Synthesis Procedures

#### Starting Materials

All chemicals and solvents if not otherwise mentioned were purchased from chemical companies and were reagent-grade. They were used without further purification or drying. All reactions were carried out under ambient conditions. All measurements were carried out on powdered samples of the respective polycrystalline compound.

#### Synthesis and Characterization of Complexes **1**–**5**

##### Complex [MnL_1_]ClO_4_ (**1**)

H_2_L_1_ (4-methoxy-Sal_2_323) was synthesized by mixing 4-methoxylsalicylaldehyde (0.076 g 0.5 mmol) with 1,2-bis(3-aminopropyl-amino)ethane (0.044 mg, 0.25 mmol), in 1:1 ethanol/acetonitrile (10.0 mL). The ligand solution was stirred for 1 h under ambient conditions to complete the Schiff base reaction and was then used directly without further purification. The ligand solution of H_2_L_1_ was added to a solution of Mn(ClO_4_)_2_·6H_2_O (0.097 g, 0.25 mmol) in 1:1 ethanol/acetonitrile (10 mL). The solution turned dark red (almost black) and was stirred for 10 min at room temperature (rt). Any precipitate was filtered off afterwards, and the reaction was left for slow evaporation. After a few days, small dark red block-shaped crystals were isolated by filtration. Mass spectrometry (g/mol): expected: 495.18 (100% complex cation). Found: 495.02. Elemental analysis for **1**, [C_24_H_32_N_4_O_4_Mn]^+^[ClO_4_]^−^ (%): calculated: C: 48.45; H: 5.42; N: 9.42; Cl: 5.96. Found: C: 48.22; H: 5.39; N: 9.30; Cl: 5.78.

##### Complex [MnL_1_]BF_4_ (**2**)

The ligand solution of H_2_L_1_ was added to a solution of MnCl_2_·4H_2_O (0.046 g, 0.25 mmol) dissolved in 1:1 ethanol/acetonitrile (10 mL) together with NH_4_BF_4_ (0.030 g, 0.3 mmol). The solution turned dark red (almost black) and was stirred for 10 min at rt. Any precipitate was filtered off afterwards, and the reaction was left for slow evaporation. After a few days, small dark red–purple thin plates of crystals were isolated by filtration. Mass spectrometry (g/mol): expected: 495.18 (100% complex cation). Found: 495.05. Elemental analysis for **2**, [C_24_H_32_N_4_O_4_Mn]^+^[BF_4_]^−^ (%): calculated: C: 49.50; H: 5.54; N: 9.62; F: 13.05. Found: C: 49.41; N: 5.49; N: 9.63; F: 13.38.

##### Complex [MnL_1_]NO_3_ (**3**)

The synthesis procedure for complex **3** is analogous to that of **1** except that 0.25 mmol of Mn(NO_3_)_2_·6H_2_O (0.055 g) was used instead of manganese(II) perchlorate hexahydrate. After a few days, small dark red plates of crystals were isolated by filtration. Mass spectrometry (g/mol): expected: 495.18 (100% complex cation). Found: 495.27. Elemental analysis for **3**, [C_24_H_32_N_4_O_4_Mn]^+^[NO_3_]^−^ (%): calculated: C: 51.71; H: 5.79; N: 12.56. Found: C: 51.64; H: 5.77; N: 12.46. The phase purity of **3** was determined by powder X-ray analysis (Supporting Information, section S3).

##### Complex [MnL_1_]Br (**4**)

The synthesis procedure for complex **4** is analogous to that of **1** except that 0.25 mmol of MnBr_2_ (0.054 g) was used instead of manganese(II) perchlorate hexahydrate. After a few days, small dark red prismatic shaped crystals were isolated by filtration. Mass spectrometry (g/mol): expected: 495.18 (100% complex cation). Found: 495.02. Elemental analysis for **4**, [C_24_H_32_N_4_O_4_Mn]^+^[Br]^−^ (%): calculated: C: 50.10; H: 5.61; N: 9.74. Found: C: 49.70; H: 5.55; N: 9.57.

##### Complex [MnL_1_]I (**5**)

The ligand solution of H_2_L_1_ was added to a solution of MnCl_2_·4H_2_O (0.046 g, 0.25 mmol dissolved in 1:1ethanol/acetonitrile (10 mL) together with NaI (0.045 g, 0.3 mmol). The solution turned dark red (almost black) and was stirred for 10 min at rt. Any precipitate was filtered off afterwards, and the reaction was left for slow evaporation. After a few days, small dark red–purple thin plates of crystals were isolated by filtration. Elemental analysis for **5**, [C_24_H_32_N_4_O_4_Mn]^+^[I]^−^ (%): calculated: C: 46.32; H: 5.18; N: 9.00. Found: C: 46.34; N: 5.12; N: 8.85.

### Crystallography

#### Crystal Data Collection and Refinement

Suitable single crystals of complexes **1**–**5** were mounted on an Oxford Diffraction Supernova A diffractometer fitted with an Atlas detector; data sets were measured using monochromatic Cu Kα radiation or Mo Kα radiation and corrected for absorption.^139^ The temperature (100 K) was controlled with an Oxford Cryosystem instrument. Structures were solved by direct methods (SHELXS)^140^ and refined with full-matrix least-squared procedures based on *F*^2^, using SHELXL-2016. Non-hydrogen atoms were refined with independent anisotropic displacement parameters; organic H atoms, i.e., bound to C or N or OH groups from methanol, were placed in idealized positions. Not all hydrogen atoms of disordered solvents could be located. The hydrogen atoms of water molecules that could be located were first located in the difference Fourier map. In subsequent refinements the O–H bond lengths (0.84 Å) and H···H distances (1.33 Å) were restrained to their ideal values. After convergence, the water molecules were refined as rigid groups. Selected crystallographic data and structure refinements are summarized in Table S3, and crystallographic data for the structures reported in this paper have been deposited with the Cambridge Crystallographic Data Centre as supplementary publication numbers CCDC 2105399–2105406. Copies of the data can be obtained free of charge from https://www.ccdc.cam.ac.uk/structures/.

## Supporting Information Available

The SI contains (i) synthesis details for compound **6**, (ii) powder X-ray analysis on complex **3**, [MnL_1_]NO_3_, (iii) temperature dependence of the χ_M_*T* products at 1000 Oe for compound **3**, [MnL_1_]NO_3_, in cooling and warming mode, (iv) the first derivative of χ_M_*T* against the temperature, simulation of χ*T* using the parameters observed from HFEPR for **5**, (v) experimental resonance fields observed for **2** and assigned to the *S* = 2 state and to the *S* = 1 state, (vi) experimental HFEPR spectrum of **2** recorded at 5 K at a microwave frequency of 633 GHz, (vii) packing arrangement of compound **5**, (viii) additional information about the DFT calculations, (ix) experimental and calculated donor bond lengths (Å) in complex **4**, (x) individual contributions to the -tensor, (xi) hydrogen bonding network found within complexes **1** and **3**–**5**, (xii) CD spectra of all single crystals of complex **1**, (xiii) solid-state (Nujol mull) UV–vis spectra of complexes **1** and **5** at room temperature, (xiv) solution UV–vis spectroscopy of the ligand H_2_L_1_, (xv) crystallographic details for compounds **1**–**6** (PDF)

## Accession Codes

CCDC 2105399–2105406 contain the supplementary crystallographic data for this paper. These data can be obtained free of charge via www.ccdc.cam.ac.uk/data_request/cif, or by emailing data_request@ccdc.cam.ac.uk, or by contacting The Cambridge Crystallographic Data Centre, 12 Union Road, Cambridge CB2 1EZ, UK; fax: +44 1223 336033.
