# Supplementary material for: Homochiral Mn3+ Spin-Crossover Complexes: A Structural and Spectroscopic Study
Source: Inorg Chem. 2022 Feb 17;61(8):3458–71. doi: 10.1021/acs.inorgchem.1c03379 (PMC8889584; doi:10.1021/acs.inorgchem.1c03379)
Supplement: Supplementary file 1 — ic1c03379_si_001.pdf [file ic1c03379_si_001.pdf]

Supporting Information

Homochiral Mn^{3+} Spin Crossover Complexes - A Structural and Spectroscopic Study

Irina A. Kühne,[a,b] Andrew Ozarowski,[c,*] Aizuddin Sultan,[a] Kane Esien,[d] Anthony B. Carter,[a] Paul Wix,[e] Aoife Casey,[a] Moonerah Heerah-Booluck,[a] Tony D. Keene,[a] Helge Müller-Bunz,[a] Solveig Felton,[d] Stephen Hill[c,f,*] and Grace G. Morgan[a,*]

Corresponding Authors
* Grace G. Morgan - grace.morgan@ucd.ie
* Andrew Ozarowski - ozarowsk@magnet.fsu.edu
* Stephen Hill - shill@magnet.fsu.edu

Index

S1. Synthetic Details for Compound **6**......3

Figure S1. Temperature dependence of the χ_MT products at 1000 Oe for compound **3**, $[MnL_1]NO_3$, in cooling and warming mode (left), and the first derivative of χ_MT against the temperature (right). 4

Figure S2. DC magnetic susceptibility of a polycrystalline sample of **4** measured at 1000 Oe in cooling mode, and simulation of χT using the parameters observed from HFEPR, *D* = +23 cm^{-1}. 4

Figure S3. Black circles: Experimental resonance fields observed for **2** and assigned to the *S* = 2 state. Purple circles: resonances assigned to the *S* = 1 state. The green, blue and red lines were calculated for the *x*, *y* and *z* orientations, respectively, using *S* = 2 with g_x = 1.972, g_y = 1.978, g_z = 1.94, *D* = +7.17 cm^{-1} and *E* = +0.17 cm^{-1}. 5

Figure S4. Black circles: Experimental resonance fields observed for **2** and assigned to the *S* = 2 state. Purple circles: resonances assigned to the *S* = 1 state. The green, blue and red lines were calculated for the *x*, *y* and *z* orientations, respectively, using *S* = 2 with g_x = 1.972, g_y = 1.978, g_z = 1.94, *D* = +6.87 cm^{-1} and *E* = +0.63 cm^{-1}. 5

Figure S5. Black circles: Experimental resonance fields observed for **2** and assigned to the *S* = 2 state. Purple circles: resonances assigned to the *S* = 1 state. The green, blue and red lines were calculated for the *x*, *y* and *z* orientations, respectively using *S* = 1 with g_x = 2.03, g_y = 2.16, g_z = 2, *D* = +23.8 cm^{-1} and *E* = +3.1 cm^{-1}. 6

Figure S6. Blue: Experimental HFEPR spectrum of **2** recorded at 5 K at a microwave frequency of 633 GHz. Red: An *S* = 2 spectrum simulated with g_x = 1.972, g_y = 1.978, g_z = 1.94, *D* = +6.87 cm^{-1} and *E* = +0.63 cm^{-1}. Green: An *S* = 1 spectrum simulated with g_x = 2.03, g_y = 2.16, g_z = 2, *D* = +23.8 cm^{-1} and *E* = +3.1 cm^{-1}. 6

S2. DFT calculations 7

Table S1. Experimental and calculated donor bond lengths (Å) in Complex **4**......7

Table S2. Individual contributions to the $\boldsymbol{D}$-tensor......7

Figure S7. Packing arrangement of compound **5** as a representative for the isotypical complexes **1** - **5**, view along the a-axis (left) and along the b-axis (right) (H atoms omitted for clarity). 8

Figure S8. Hydrogen bonding network formed between the hydrogen atoms of the organic Schiff base ligand of four cationic species with one perchlorate anion of complex **1**. 8

Figure S9. Hydrogen bonding network formed between the hydrogen atoms of the organic Schiff base ligand of four cationic species with one nitrate anion of complex **3** at 100 K (left) and at room temperature (right). 8

Figure S10. Short H-Br bonds formed between the hydrogen atoms of the organic Schiff base ligand of three cationic species with one bromide anion of complex **4** at 100 K (left) and at 190 K (right). 9

Figure S11. Short H-I bonds formed between the hydrogen atoms of the organic Schiff base ligand of three cationic species with one iodide anion of complex **5** at 100 K. 9

Figure S12. CD spectra of the first 10 single crystals of complex **1**, $[MnL_1]ClO_4$, with 3:7 ratio (green : red/purple). 10

Figure S13. CD spectra of the second set of 10 single crystals of complex **1**, $[MnL_1]ClO_4$, with a 4:6 ratio (green : red/purple). 10

Figure S14. CD spectra of the third set of 10 single crystals of complex **1**, $[MnL_1]ClO_4$, with a 3:7 ratio (green : red/purple). 11

Figure S15. Solid-state (Nujol mull) UV-Vis spectra of complexes **1** and **5** at room temperature. 11

Figure S16. Solution UV-Vis spectroscopy of the ligand H_2L_1 recorded in acetonitrile solution, between 200 - 350 nm. 12

Figure S17. Solution UV-Vis spectroscopy of complexes **1** and **5** recorded in methanol solution, between 200 - 800 nm (~1.5×10^{-5} M). Inset: zoom between 400 - 800 nm. 12

S3. Powder X-ray analysis on complex **3** $[MnL_1]NO_3$. 13

Table S3. Crystallographic Details for Compounds **1** - **6**...14

S4. References 16

S1. Synthetic Details for Compound **6**

All chemicals and solvents if not otherwise mentioned were purchased from chemical companies and were reagent grade. They were used without further purification or drying. All reactions were carried out under ambient conditions. All measurements were carried out on a powdered samples of the polycrystalline compound.

Synthesis and characterization of complex 6.

Complex [MnL]Cl·0.34MeOH·3.93H$_2$O (6). H$_2$L (4-methoxy-sal$_2$323) was synthesised starting with the condensation of 4-methoxylsalicylaldehyde (0.076 g, 0.5 mmol) with 1,2-bis(3-aminopropyl-amino)ethane (0.044 mg 0.25 mmol) in 1:1 ethanol/acetonitrile (10.0 ml). The ligand solution was stirred for one hour under ambient conditions to complete the Schiff base reaction and was then used directly without further purification. The ligand solution of H$_2$L$_1$ was added to a solution of MnCl$_2$·4H$_2$O (0.050 g, 0.25 mmol) dissolved in 1:1 ethanol/acetonitrile (10 ml). The solution turned dark red (almost black) and was stirred for 10 minutes at r.t. Any precipitate was filtered off and the filtrate was left for slow evaporation. After several days, small dark red block-shaped crystals were isolated.

The structure of the cationic [MnL$_1$]$^+$ species of (**6**) is shown below together with a table of core bond lengths.

bond lengths of (**6**) in Å	
Mn–O(1)	1.8875(17)
Mn–O(4)	1.9076(17)
Mn–N(1)	2.078(2)
Mn–N(4)	2.110(2)
Mn–N(3)	2.189(2)
Mn–N(2)	2.197(2)

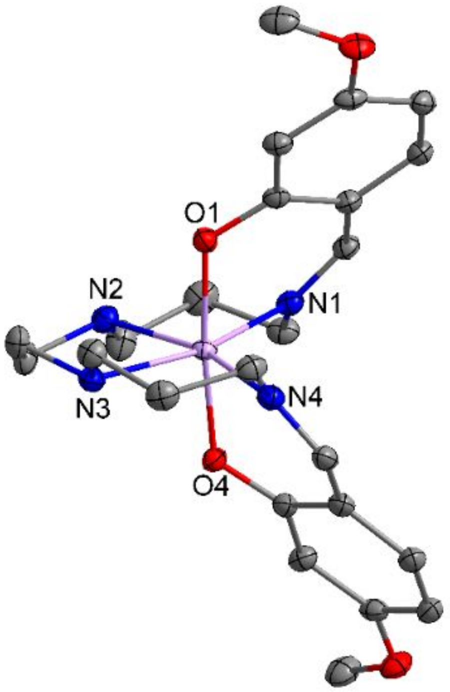

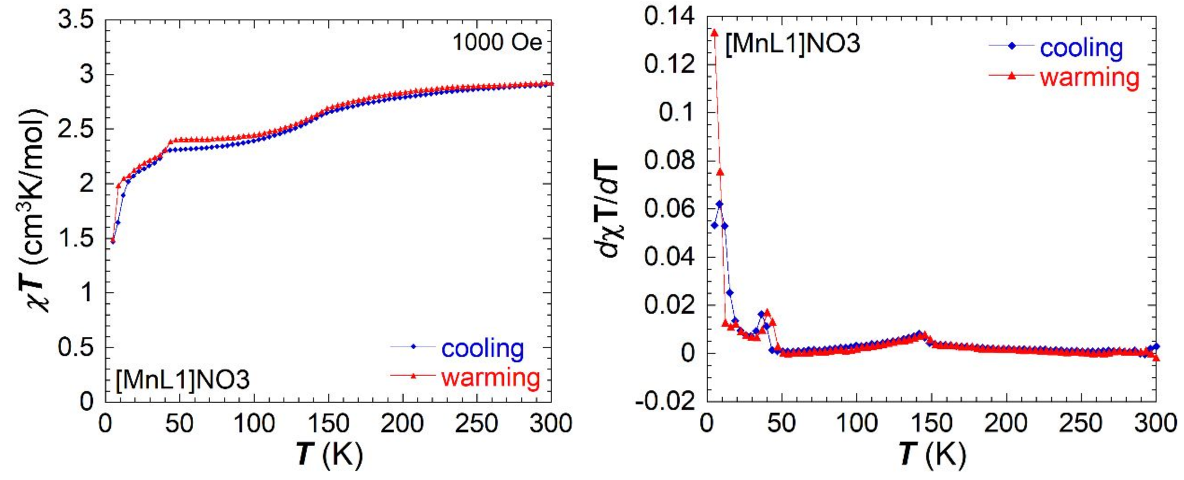

Figure S1. Temperature dependence of the $\chi_M T$ products at 1000 Oe for compound **3**, $[MnL_1]NO_3$, in cooling and warming mode (left), and the first derivative of $\chi_M T$ against the temperature (right).

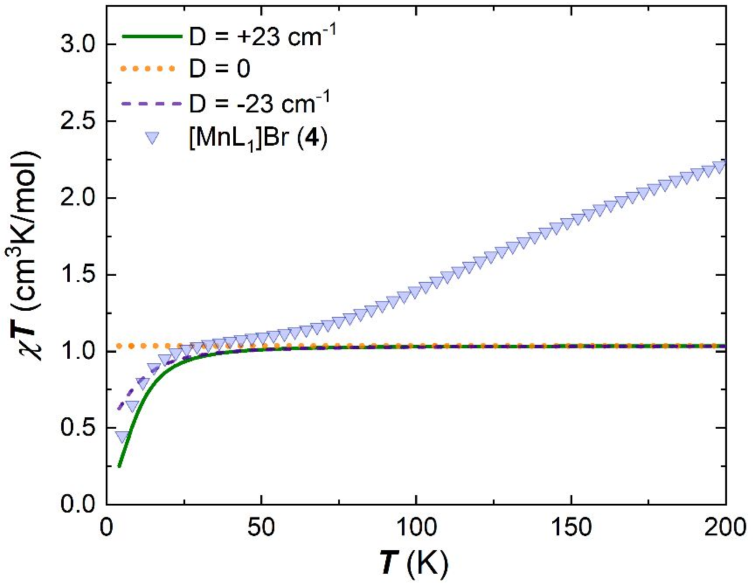

Figure S2. DC magnetic susceptibility of a polycrystalline sample of **4** measured at 1000 Oe in cooling mode, and simulation of χT using the parameters observed from HFEPR, $D = +23\ cm^{-1}$. Experimental points are shown as pale blue triangles together with simulations using the parameters from the HFEPR experiment, as solid green line. For comparison $D = 0$ and $D = -23\ cm^{-1}$ are shown as dotted orange line and dashed purple line, respectively. Simulations were performed using the EasySpin software package[1] for Matlab.

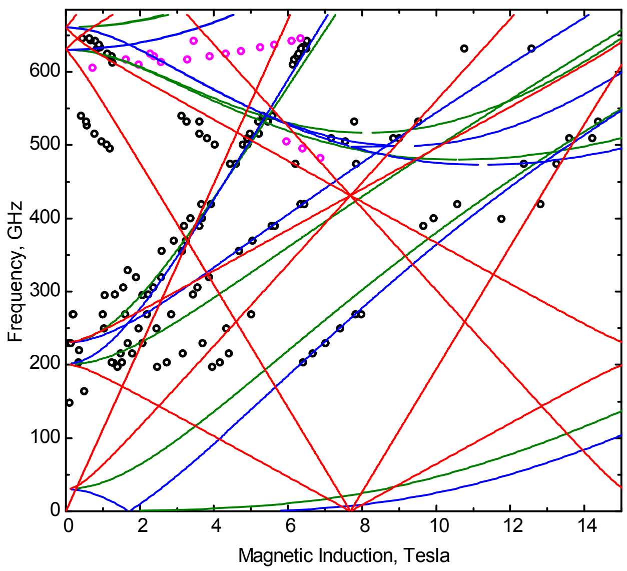

Figure S3. Black circles: Experimental resonance fields observed for **2** and assigned to the *S* = 2 state. Purple circles: resonances assigned to the *S* = 1 state. The green, blue and red lines were calculated for the *x*, *y* and *z* orientations, respectively, using *S* = 2 with g_x = 1.972, g_y = 1.978, g_z = 1.94, *D* = +7.17 cm^{-1} and *E* = +0.17 cm^{-1}.

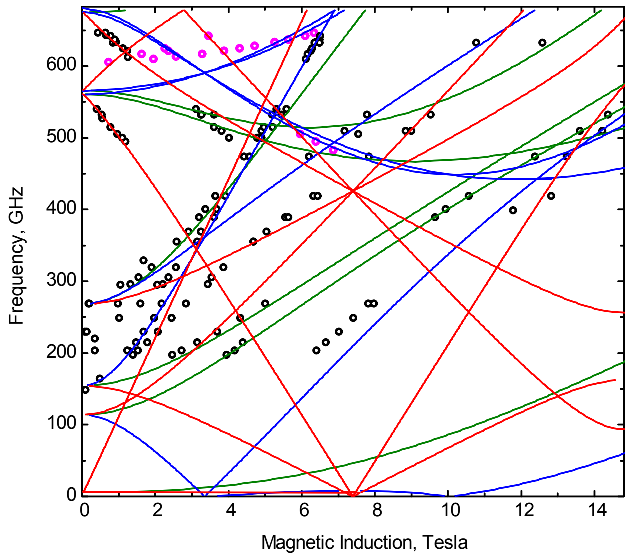

Figure S4. Black circles: Experimental resonance fields observed for **2** and assigned to the *S* = 2 state. Purple circles: resonances assigned to the *S* = 1 state. The green, blue and red lines were calculated for the *x*, *y* and *z* orientations, respectively, using *S* = 2 with g_x = 1.972, g_y = 1.978, g_z = 1.94, *D* = +6.87 cm^{-1} and *E* = +0.63 cm^{-1}.

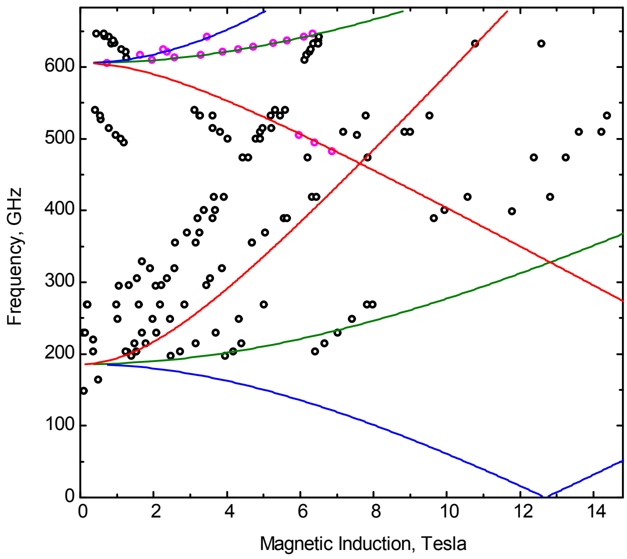

Figure S5. Black circles: Experimental resonance fields observed for **2** and assigned to the *S* = 2 state. Purple circles: resonances assigned to the *S* = 1 state. The green, blue and red lines were calculated for the *x*, *y* and *z* orientations, respectively using *S* = 1 with g_x = 2.03, g_y = 2.16, g_z = 2, *D* = +23.8 cm^{-1} and *E* = +3.1 cm^{-1}.

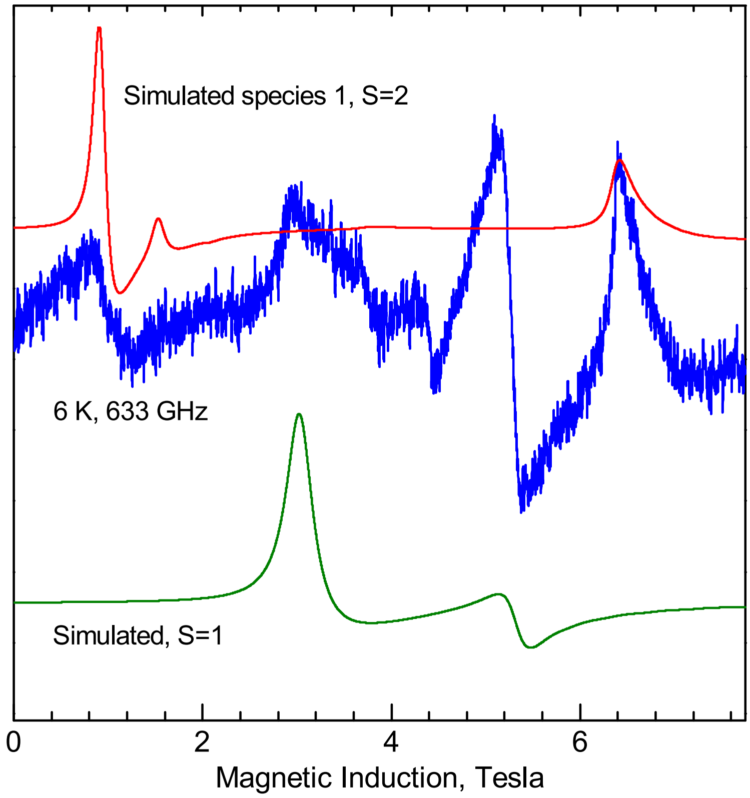

Figure S6. Blue: Experimental HFEPR spectrum of **2** recorded at 5 K at a microwave frequency of 633 GHz. Red: An *S* = 2 spectrum simulated with g_x = 1.972, g_y = 1.978, g_z = 1.94, *D* = +6.87 cm^{-1} and *E* = +0.63 cm^{-1}. Green: An *S* = 1 spectrum simulated with g_x = 2.03, g_y = 2.16, g_z = 2, *D* = +23.8 cm^{-1} and *E* = +3.1 cm^{-1}.

S2. DFT calculations

The optimization preserves the molecular symmetry resulting in equal Mn-O bonds and two pairs of equal Mn-N bonds.

Table S1. Experimental and calculated donor bond lengths (Å) in Complex **4**.

Bond	X-Ray	S = 1	S = 2
Mn-O	1.8994	1.88	1.876
Mn-N	2.0114	1.951	2.071
Mn-N	2.0847	2.081	2.289

The S=1 state is favored by 32.3 kJ/mol compared to the S=2 state.

Calculation of *D*, *E* and *g* in 2, using the optimized structure for *S* = 2:

D = +3.84 cm^{-1}, E = +0.45 cm^{-1} (spin-orbit and spin-spin contributions)

g_x = 1.974, g_y = 1.984, g_z = 1.986

Table S2. Individual contributions to the $\overleftrightarrow{\boldsymbol{D}}$-tensor.

Multiplicity	Energy, cm^{-1}	D, cm^{-1}	E, cm^{-1}
5	10584	-0.039	-0.000
5	17502	0.670	0.670
5	20953	0.445	-0.449
5	22114	0.036	0.036
3	14507	2.209	0.008
3	15870	-0.248	-0.248
3	17763	-0.105	0.209
3	20953	-0.001	-0.001
3	22114	-0.139	0.171
3	25856	-0.017	-0.017
3	26336	0.066	0.003
3	26537	-0.041	-0.041
3	28678	-0.004	-0.004
3	29907	0.467	0.038

The largest contribution comes from a triplet state (in red).

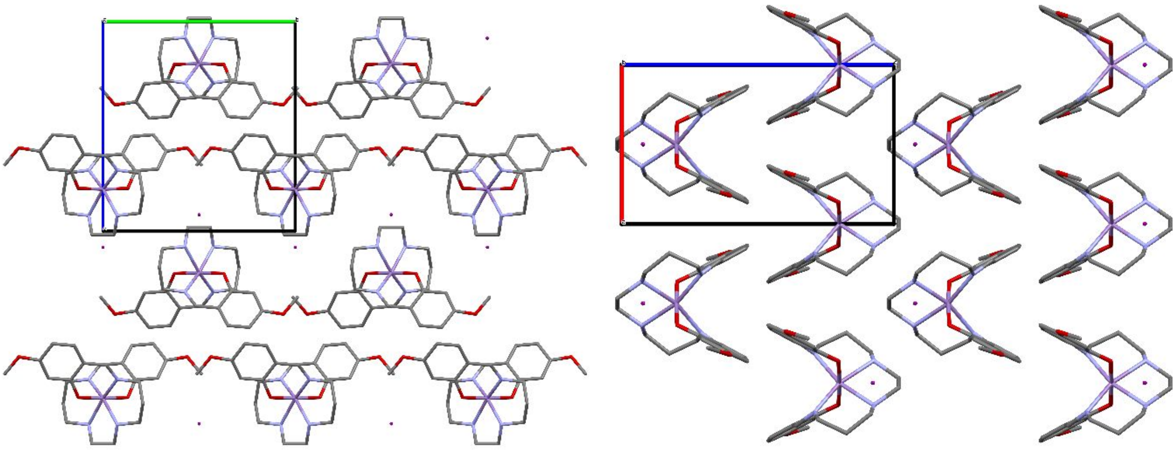

Figure S7. Packing arrangement of compound **5** as a representative for the isotypical complexes **1** - **5**, view along the a-axis (left) and along the b-axis (right) (H atoms omitted for clarity).

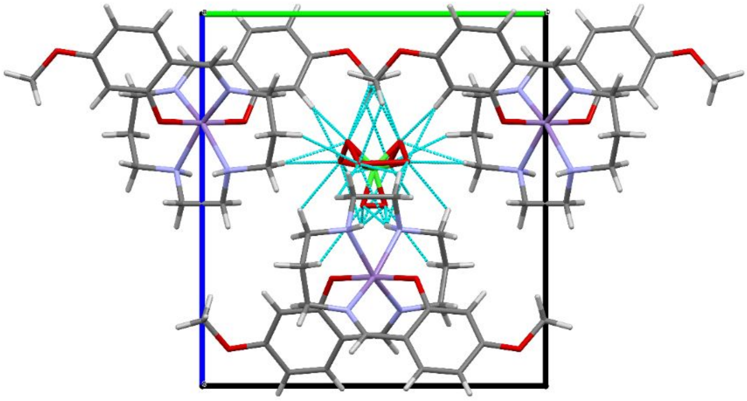

Figure S8. Hydrogen bonding network formed between the hydrogen atoms of the organic Schiff base ligand of four cationic species with one perchlorate anion of complex **1**.

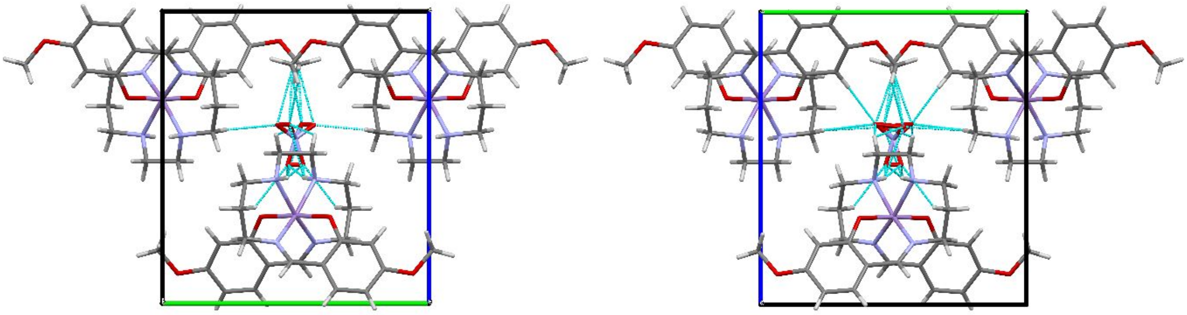

Figure S9. Hydrogen bonding network formed between the hydrogen atoms of the organic Schiff base ligand of four cationic species with one nitrate anion of complex **3** at 100 K (left) and at room temperature (right).

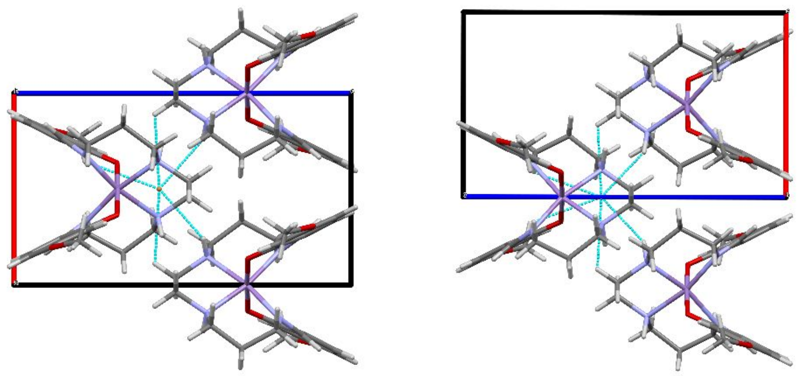

Figure S10. Short H-Br bonds formed between the hydrogen atoms of the organic Schiff base ligand of three cationic species with one bromide anion of complex **4** at 100 K (left) and at 190 K (right).

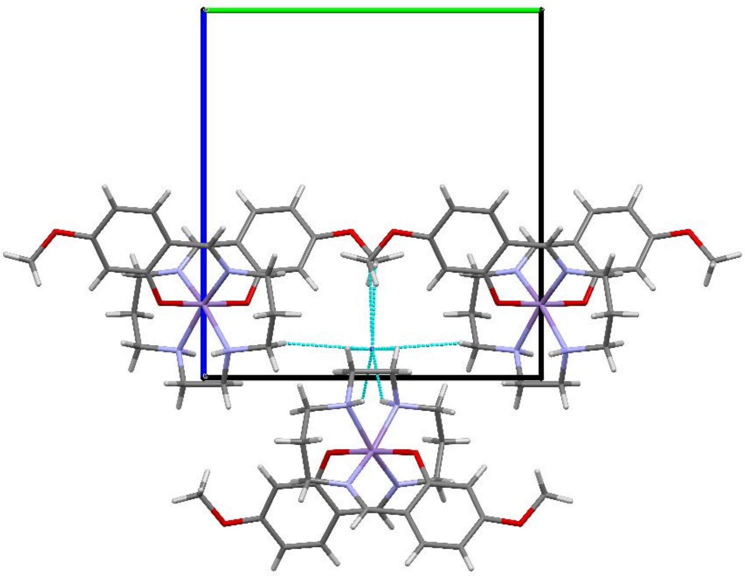

Figure S11. Short H-I bonds formed between the hydrogen atoms of the organic Schiff base ligand of three cationic species with one iodide anion of complex **5** at 100 K.

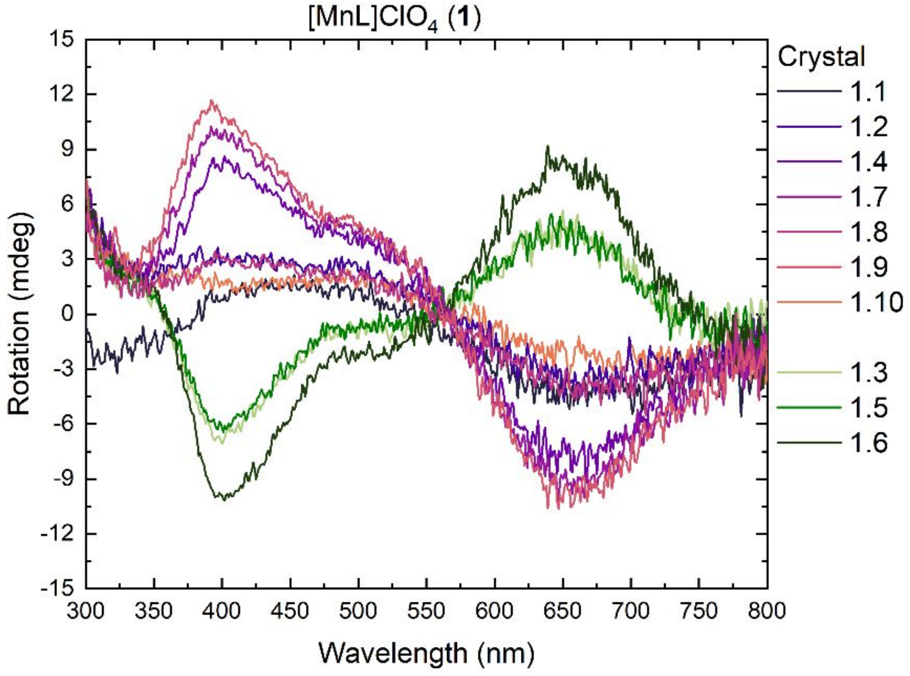

Figure S12. CD spectra of the first 10 single crystals of complex **1**, $[MnL_1]ClO_4$, with 3:7 ratio (green : red/purple).

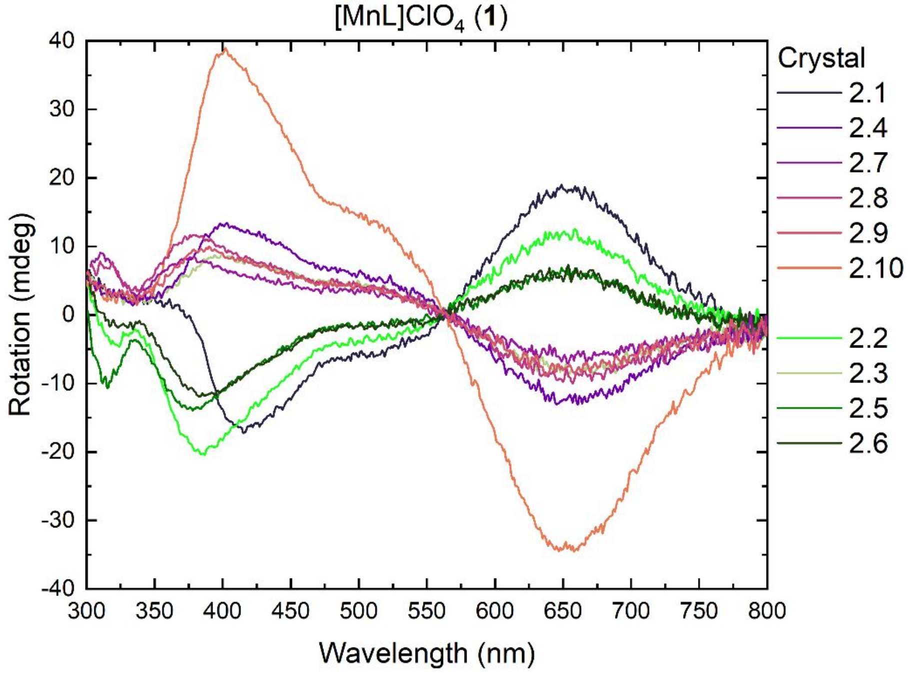

Figure S13. CD spectra of the second set of 10 single crystals of complex **1**, $[MnL_1]ClO_4$, with a 4:6 ratio (green : red/purple).

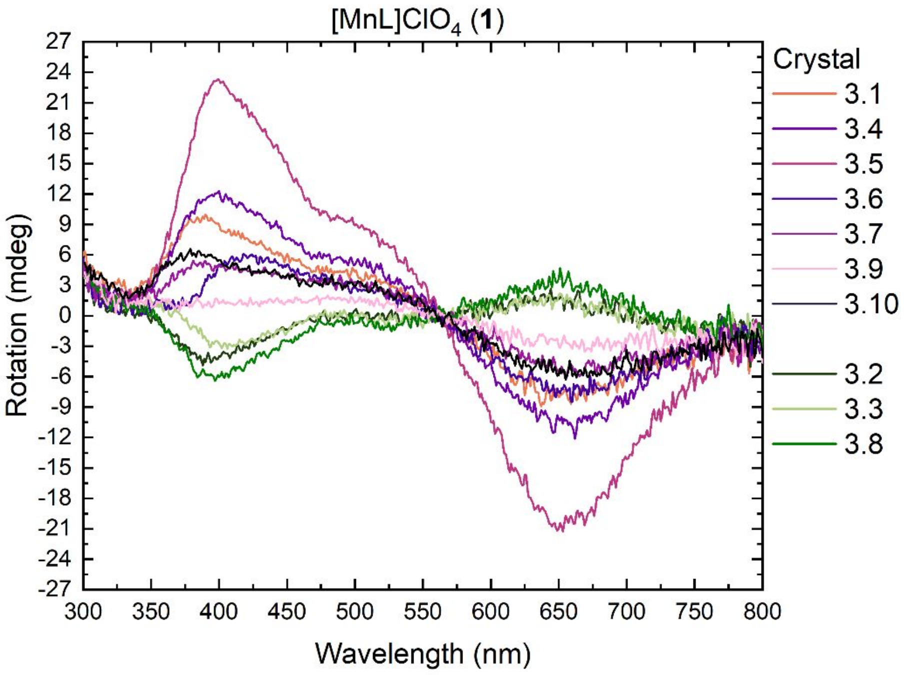

Figure S14. CD spectra of the third set of 10 single crystals of complex **1**, $[MnL_1]ClO_4$, with a 3:7 ratio (green : red/purple).

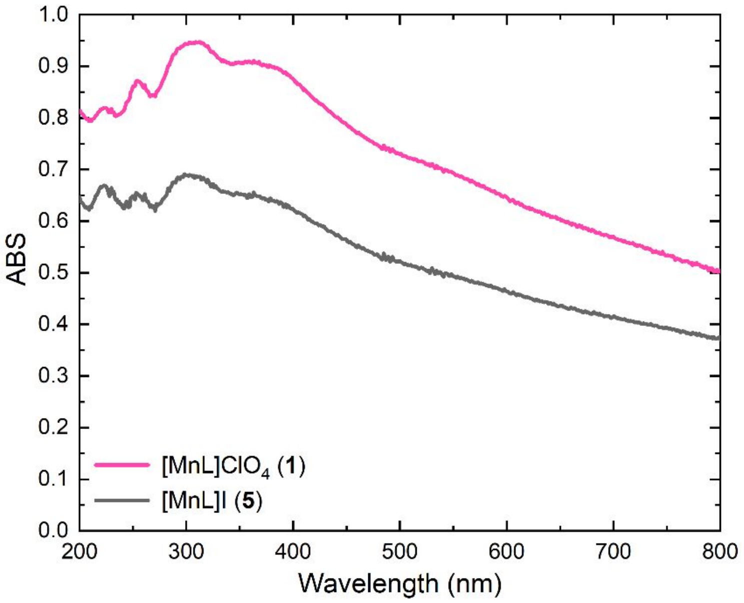

Figure S15. Solid-state (Nujol mull) UV-Vis spectra of complexes **1** and **5** at room temperature.

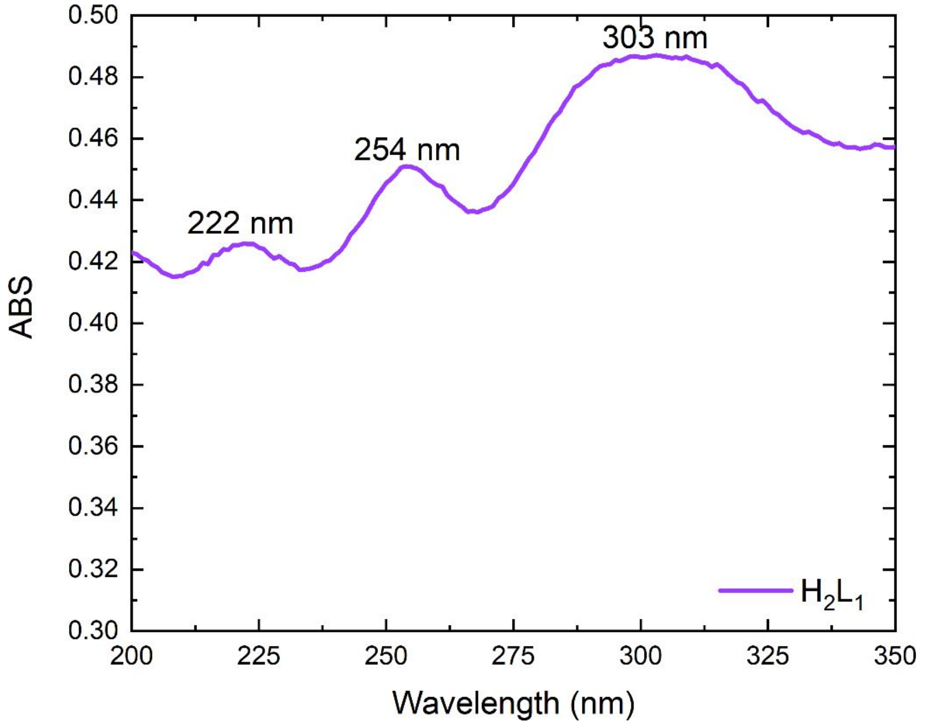

Figure S16. Solution UV-Vis spectroscopy of the ligand H_2L_1 recorded in acetonitrile solution, between 200 - 350 nm.

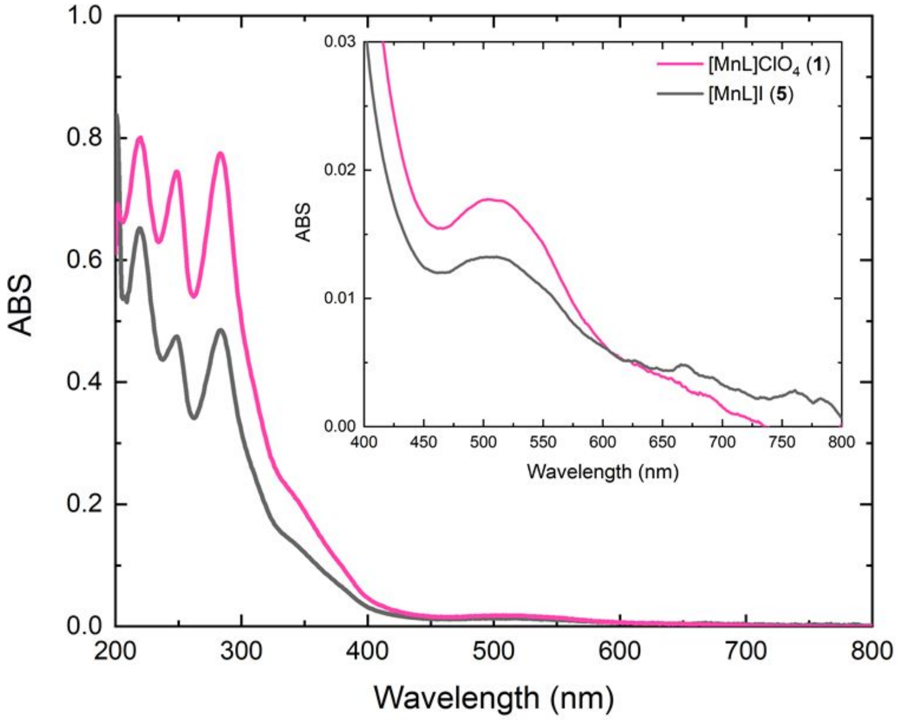

Figure S17. Solution UV-Vis spectroscopy of complexes **1** and **5** recorded in methanol solution, between 200 - 800 nm (~1.5×10^{-5} M). Inset: zoom between 400 - 800 nm.

S3. Powder X-ray analysis on complex **3** [MnL_1]NO_3.

Powder X-Ray diffraction (PXRD) experiments were carried out using a Bruker D2 Phaser with CuKα radiation λ=1.5418 Å. The sample was used as prepared and measured on a zero-background silicon sample holder. The data were collected in the 2Θ Range from 5 – 55° in 0.01° increments at room temperature while rotating the sample at one rotation per minute in the ϕ direction. Background due to fluorescence was subtracted. The pattern indicated a preferred orientation due to the sample consisting of thin plates. To account for this the patterns were compared to calculated patterns with a preferred orientation of h,k,l=1,0,-1 and a March-Dollase parameter of 0.7. Simulations were carried out with Mercury.

Powder diffraction pattern of a dried sample of complex **3** (top black) is shown below and is compared with the theoretical powder pattern calculated from the cif-file of the room temperature structure of **3** (mor1162-RT) (bottom red), highlighting the purity of the sample.

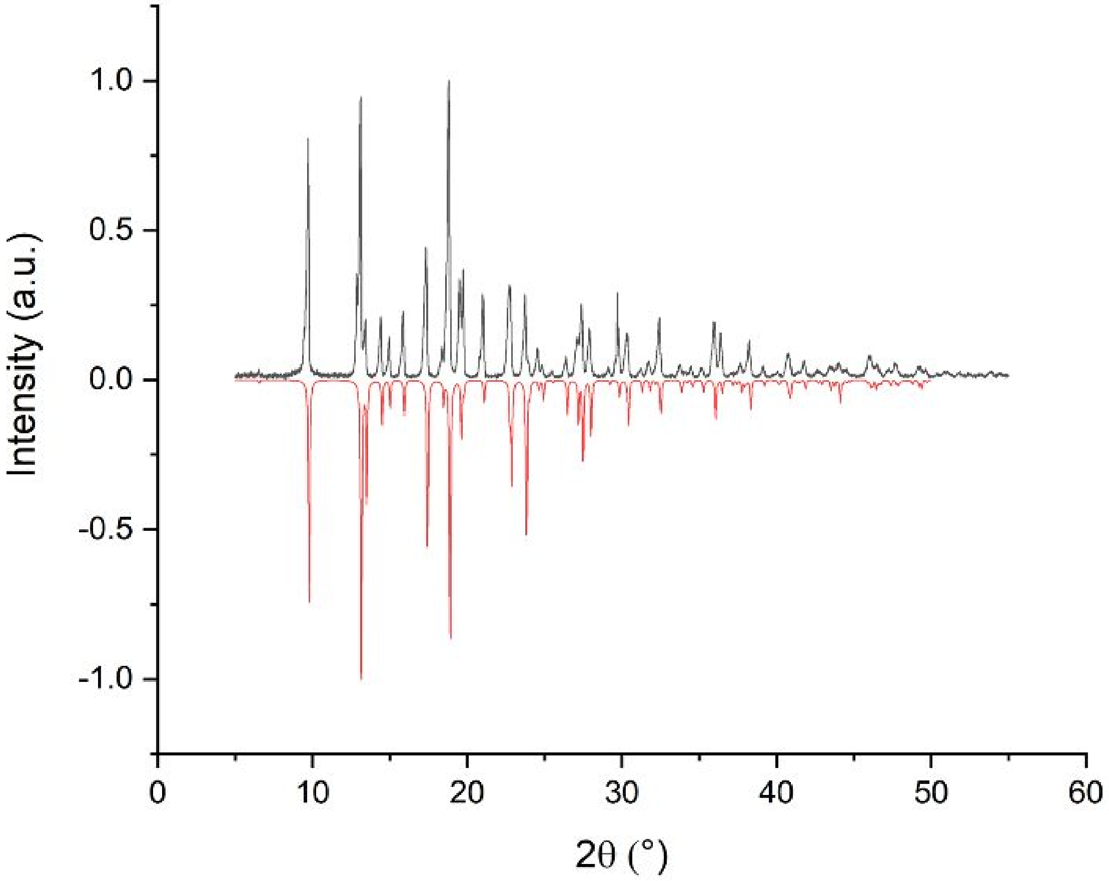

Table S3. Crystallographic Details for Compounds **1** - **6**.

Compound	$[MnL_1]ClO_4$ (**1**)	$[MnL_1]BF_4$ (**2**)	$[MnL_1]NO_3$ (**3**)
sample code	mor731 (100K)	2017tdk043 (100K)	mor730 (100K)
Empirical formula	$C_{24}H_{32}N_4O_8ClMn$	$C_{24}H_{32}BN_4O_4F_4Mn$	$C_{24}H_{32}N_5O_7Mn$
Formula weight	594.93	582.28	557.49
Radiation	Cu-Kα	Mo-Kα	Cu-Kα
Temperature (K)	100(2)	100(2)	100(2)
Crystal system	orthorhombic	orthorhombic	orthorhombic
Space group	$P2_12_12$	$P2_12_12$	$P2_12_12$
Crystal size (mm)	0.1537 x 0.1301 x 0.0803	0.18 x 0.13 x 0.01	0.1736 x 0.1170 x 0.0659
a (Å)	7.9644(1)	7.8070(2)	7.6362(1)
b (Å)	12.35783(12)	12.2709(4)	12.14089(13)
c (Å)	13.36722(14)	13.4111(4)	13.30789(14)
α (°)	90	90	90
β (°)	90	90	90
γ (°)	90	90	90
V (Å^3)	1315.64(3)	1284.77(7)	1233.78(2)
Z	2	2	2
d_{calc} (g cm^{-3})	1.502	1.505	1.501
μ (mm^{-1})	0.659	0.582	4.826
F(000)	620	604	584
Limiting indices	h = ± 12, k = ± 19, l = ± 19	h = ± 9, k = ± 14, l = ± 15	h = ± 9, k = ± 15, l = ± 16
Reflections collected / unique	34329/4674	7260/2269	25738/2596
R(int)	0.0300	0.0363	0.0371
Completeness to Θ (%)	99.7	99.9	99.9
Data / restraints / parameters	4674 / 0 / 193	2269 / 0 / 191	2596 / 0 / 171
GooF on F^2	1.117	1.137	1.049
Final R indices [$I > 2\sigma(I)$][a]	R_1 = 0.0259, wR_2 = 0.0641	R_1 = 0.0399, wR_2 = 0.0754	R_1 = 0.0417, wR_2 = 0.1051
R indices (all data)	R_1 = 0.0302, wR_2 = 0.0668	R_1 = 0.0440, wR_2 = 0.0771	R_1 = 0.0421, wR_2 = 0.1054
Largest diff. peak/hole (e·Å^{-3})	0.324 and -0.280	0.259 and -0.299	0.853 and -0.508
CCDC no.	2105401	2105399	2105400

Compound	$[MnL_1]NO_3$ (**3**)	$[MnL_1]Br$ (**4**)	$[MnL_1]Br$ (**4**)
sample code	mor1162 (RT)	mor1370 (100K)	mor1302 (190 K)
Empirical formula	$C_{24}H_{32}N_5O_7Mn$	$C_{24}H_{32}BrMnN_4O_4$	$C_{24}H_{32}BrMnN_4O_4$
Formula weight	557.48	575.38	575.38
Temperature (K)	293(2)	100(2)	190(2)
Radiation	Cu-Kα	Mo-Kα	Mo-Kα
Crystal system	orthorhombic	orthorhombic	orthorhombic
Space group	$P2_12_12$	$P2_12_12$	$P2_12_12$
Crystal size (mm)	0.191 x 0.116 x 0.084	0.323 x 0.246 x 0.123	0.303 x 0.177 x 0.124
a (Å)	7.76661(7)	7.45865(9)	7.5238(1)

b (Å)	12.20894(9)	12.1055(2)	12.1201(2)
c (Å)	13.4495(1)	13.1032(2)	13.1574(2)
α (°)	90	90	90
β (°)	90	90	90
γ (°)	90	90	90
V (Å^3)	1275.309(18)	1183.10(3)	1199.81(3)
Z	2	2	2
d_{calc} (g cm^{-3})	1.452	1.615	1.593
μ (mm^{-1})	4.669	2.286	2.254
F(000)	584	592	592
Limiting indices	h = ± 9, k = ± 15, l = ± 16	h = ± 11, k = ± 18, l = ± 19	h = ± 11, k = ± 18, l = ± 20
Reflections collected / unique	13225/2669	75846/4288	19471/4200
R(int)	0.0241	0.0394	0.0266
Completeness to Θ (%)	100.0	99.8	99.8
Data / restraints / parameters	2669 / 0 / 171	4288 / 0 / 160	4200 / 0 / 156
GooF on F^2	1.026	1.040	1.023
Final R indices [I > 2σ(I)][a]	R_1 = 0.0378, wR_2 = 0.1055	R_1 = 0.0198, wR_2 = 0.0458	R_1 = 0.0257, wR_2 = 0.0591
R indices (all data)	R_1 = 0.0387, wR_2 = 0.1071	R_1 = 0.0227, wR_2 = 0.0472	R_1 = 0.0299, wR_2 = 0.0611
Largest diff. peak/hole (e·Å^{-3})	0.399 and -0.408	0.419 and -0.292	0.505 and -0.340
CCDC no.	2105402	2105405	2105403

Compound	[MnL$_1$]I (**5**)	[MnL$_1$]Cl·0.3MeOH·4 H_2O (**6**)
sample code	mor1362 (100 K)	mor1467 (100K)
Empirical formula	C_{24} H_{32} N_4 O_4 Mn I	$C_{24.34}$ $H_{41.22}$ N_4 $O_{8.27}$ Cl Mn
Formula weight	622.37	612.58
Temperature (K)	100(2)	100(2)
Radiation	Mo-Kα	Cu-Kα
Crystal system	orthorhombic	orthorhombic
Space group	*P2₁2₁2*	*Pccn*
Crystal size (mm)	0.211 x 0.077 x 0.053	0.195 x 0.152 x 0.043
a (Å)	7.7289(3)	12.1273(1)
b (Å)	12.1498(3)	31.5117(4)
c (Å)	13.2316(4)	14.7028(2)
α (°)	90	90
β (°)	90	90
γ (°)	90	90
V (Å^3)	1245.51(7)	5618.70(11)
Z	2	8
d_{calc} (g cm^{-3})	1.664	1.448
μ (mm^{-1})	1.812	5.163
F(000)	628	2587.36

Limiting indices	h = ± 10, k = ± 16, l = ± 17	h = ± 15, k = ± 39, l = ± 18
Reflections collected / unique	15815/3180	29015/5896
R(int)	0.0766	0.0337
Completeness to Θ (%)	99.8	99.9
Data / restraints / parameters	3180 / 0 / 156	5896 / 0 / 393
GooF on F^2	1.079	1.083
Final R indices [$I > 2\sigma(I)$][a]	R_1 = 0.0432, wR_2 = 0.1004	R_1 = 0.0482, wR_2 = 0.1249
R indices (all data)	R_1 = 0.0493, wR_2 = 0.1069	R_1 = 0.0515, wR_2 = 0.1273
Largest diff. peak/hole ($e·Å^{-3}$)	1.752 and -1.574	0.686 and -0.714
CCDC no.	2105404	2105406

S4. References

(1) Stoll, S.; Schweiger, A. EasySpin, a Comprehensive Software Package for Spectral Simulation and Analysis in EPR. *J. Magn. Reson.* **2006**, *178* (1), 42–55. ttps://doi.org/10.1016/j.jmr.2005.08.013.
